# AI-based analysis of CT images for rapid triage of COVID-19 patients

**DOI:** 10.1038/s41746-021-00446-z

**Published:** 2021-04-22

**Authors:** Qinmei Xu, Xianghao Zhan, Zhen Zhou, Yiheng Li, Peiyi Xie, Shu Zhang, Xiuli Li, Yizhou Yu, Changsheng Zhou, Longjiang Zhang, Olivier Gevaert, Guangming Lu

**Affiliations:** 1 Department of Medical Imaging, Jinling Hospital, Nanjing University School of Medicine, Nanjing, Jiangsu China; 2 Stanford Center for Biomedical Informatics Research (BMIR), Department of Medicine, Stanford University, Stanford, CA USA; 3 Department of Bioengineering, Stanford University, Stanford, CA USA; 4 Deepwise AI Lab, Deepwise Inc., Beijing, China; 5 Department of Biomedical Data Science, Stanford University, Stanford, CA USA; 6 Department of Radiology, The Sixth Affiliated Hospital of Sun Yat-sen University, Guangzhou, Guangdong China

**Keywords:** Risk factors, Diseases, Signs and symptoms

## Abstract

The COVID-19 pandemic overwhelms the medical resources in the stressed intensive care unit (ICU) capacity and the shortage of mechanical ventilation (MV). We performed CT-based analysis combined with electronic health records and clinical laboratory results on Cohort 1 (*n* = 1662 from 17 hospitals) with prognostic estimation for the rapid stratification of PCR confirmed COVID-19 patients. These models, validated on Cohort 2 (*n* = 700) and Cohort 3 (*n* = 662) constructed from nine external hospitals, achieved satisfying performance for predicting ICU, MV, and death of COVID-19 patients (AUROC 0.916, 0.919, and 0.853), even on events happened two days later after admission (AUROC 0.919, 0.943, and 0.856). Both clinical and image features showed complementary roles in prediction and provided accurate estimates to the time of progression (*p* < 0.001). Our findings are valuable for optimizing the use of medical resources in the COVID-19 pandemic. The models are available here: https://github.com/terryli710/COVID_19_Rapid_Triage_Risk_Predictor.

## Introduction

From 30 December to 11 October, the ongoing severe acute respiratory syndrome–coronavirus 2 (SARS-CoV-2) pandemic has caused over 37 million coronavirus disease 2019 (COVID-19) confirmed cases and 1 million deaths globally^1^. The spread of COVID-19 continues to overwhelm medical resources without effective therapeutics and vaccines. In particular, stressed intensive care unit (ICU) capacity and the shortage of mechanical ventilation (MV) are major factors that drive COVID-19 mortality rates^2–4^. To enable sufficient supply of medical resources, rapid triage method for COVID-effected patients with potentially serious outcomes has become an urgent priority for reallocating medical resources as well as distributing patients to balance ICU loads across affected regions so as to deliver timely treatment^5–8^.

Evaluating the severity of patients with infectious pneumonia has been applied in clinics such as measuring the acute physiology and chronic health evaluation II (APACHE-II) score and laboratory indicators including neutrophil-to-lymphocyte ratio (NLR)^9–12^. However, the scoring systems of APACHE-II are highly subjective and time-consuming while laboratory indicators are not comprehensive enough to predict the adverse outcomes of the newly emerged COVID-19. Although computed tomography (CT) assessment by radiologists is now an important criterion for COVID-19 diagnosis and severity evaluation of COVID-19^13^, it is limited by manual evaluation of radiologists with marked inter- and intra-observer variability and unable to provide accurate prognosis prediction. Better ways to utilize multi-modal data for grouping hospitalized COVID-19 patients according to their potential clinical outcomes remain to be developed to deliver specific treatment timely.

In this study, we provided risk stratification based on CT-based radiomics features and clinical data for COVID-19 patients in terms of stable or severe disease (requiring ICU) on admission. Then we developed specific outcome prediction (MV/death) models for critically ill patients. Finally, we provided insights into estimating time to the progression (ICU/MV/death) for COVID-19 patients.

## Results

### Patient cohort

**Fig. 1:**
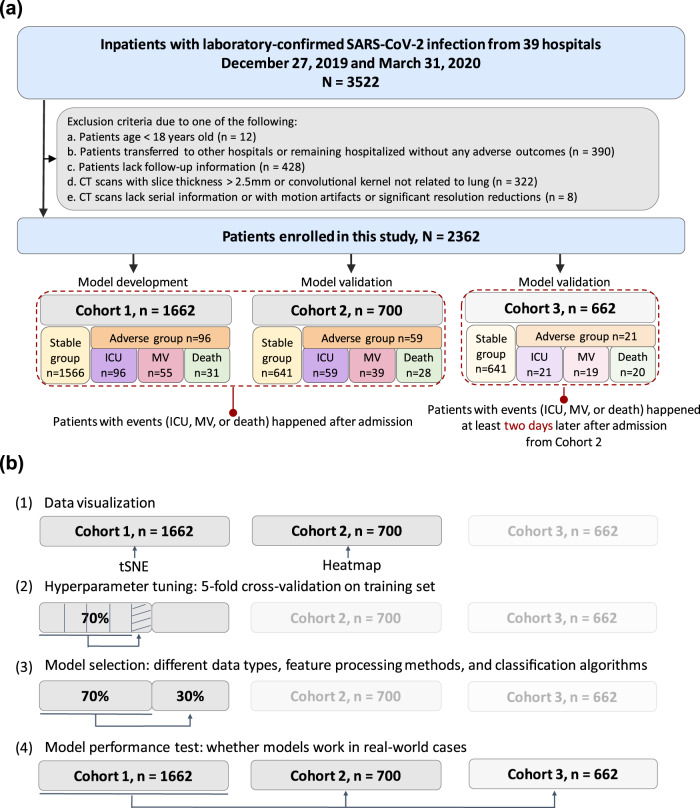
Illustration of workflow in this study. **a** Our primary cohort (Cohort 1, *n* = 1662) for model development included patients from 17 hospitals, and our validation cohort (Cohort 2, *n* = 700) consisted of patients from 7 external and independent medical centers. In addition, we built a specific cohort (Cohort 3, *n* = 662) for patients from the 7 medical centers whose interval between admission and progression to critical outcomes (ICU/MV/death) were more than two days, aiming to evaluate the performance of our models on predicting events happening at least two days after admission. **b** Explanation of our data split and the corresponding usages. (1) Step one: feature visualization of Cohort 1 and Cohort 2 to get the preliminary intuitive sense; (2) Step two: 70% samples of Cohort 1 were picked as the training set using stratified sampling based on death cases, where fivefold cross-validation was used to tune the hyperparameters of the models; (3) Step three: model selection was performed on the remaining 30% samples of Cohort 1; (4) Step four: Cohort 2 and Cohort 3 were used to evaluate model performance in different aspects.

We collected 3522 inpatients with laboratory-confirmed SARS-CoV-2 infection from December 27, 2019 to March 31, 2020, from 39 hospitals in China. Data inclusion criteria were as follows: patients received CT examination within 3 days after admission and we had definitive medical records of short-term outcomes such as intensive care unit (ICU), mechanical ventilation (MV) therapy, death (defined as the three prediction tasks), or discharge. Finally, 2362 patients were used in this study, including a primary cohort (Cohort 1, *n* = 1662) for model development, which included patients from 17 hospitals, and a validation cohort (Cohort 2, *n* = 700) which consisted of patients from nine external and independent medical centers (Fig. 1, Supplementary Table 1). In addition, we built a specific subset of Cohort 2 (Cohort 3, *n* = 662) for patients from the nine medical centers whose time intervals between admission and progression to critical outcomes (ICU/MV/death) were more than two days, aiming to evaluate the performance of our models on predicting events happening at least two days after admission. Prediction models were built for three prediction tasks, including ICU (adverse cases in Cohort 1/Cohort 2/Cohort 3, *n* = 96/59/21, respectively), MV (adverse cases in Cohort 1/2/3, *n* = 55/39/19), and death (adverse cases in Cohort 1/2/3, *n* = 31/28/20). Note that most patients with death were also in the MV group, while all patients with MV or death were in the ICU group. In our study, 2207 patients (93.5%) were discharged without any adverse outcome (stable group), 155 (6.5%) patients developed adverse clinical outcomes and were admitted to the ICU (adverse group), of whom 94 (60.6%) required MV, and 59 (38.0%) died within 28 days after admission (Table 1, Supplementary Table 2). This cohort had 1229 men (52.0%) and 1133 women (48.0%), with a median age of 51.5 years (IQR, 39–64 years). The median age among men was 57 years (IQR, 45–68 years) and the median age among women was 52 years (IQR, 39–64 years). No statistical difference in age was found between men and women in this cohort.

**Table 1:** Clinical characteristics of COVID-19 patients in Cohort 1, Cohort 2, Cohort 3, and the whole cohort.

	All patients (*n* = 2362)	Cohort 1 (*n* = 1662)	Cohort 2 (*n* = 700)	Cohort 3 (*n* = 662)	*p*-value
*Demographics*					
Age (years)	51.720 ± 15.646	52.465 ± 15.863	49.953 ± 14.984	48.985 ± 14.545	<0.001
Gender (male)	1229 (52.0%)	881 (53.0%)	348 (49.7%)	338 (51.0%)	0.143
*Comorbidity*					
Coronary heart disease	172 (7.2%)	123 (7.4%)	49 (7.0%)	36 (5.4%)	0.732
Chronic liver disease	82 (3.4%)	58 (3.4%)	24 (3.4%)	24 (3.6%)	0.941
Chronic kidney disease	29 (1.2%)	18 (1.0%)	11 (1.5%)	8 (1.2%)	0.325
COPD	51 (2.1%)	33 (1.9%)	18 (2.5%)	12 (1.8%)	0.371
Diabetes	261 (11.0%)	191 (11.4%)	70 (10.0%)	59 (8.9%)	0.291
Hypertension	500 (21.1%)	370 (22.2%)	130 (18.5%)	110 (16.6%)	0.045
Carcinoma	61 (2.5%)	44 (2.6%)	17 (2.4%)	15 (2.2%)	0.759
*Clinical symptom*					
Fever	1950 (82.5%)	1340 (80.6%)	610 (87.1%)	580 (87.6%)	<0.001
Cough	1651 (69.8%)	1170 (70.3%)	481 (68.7%)	455 (68.7%)	0.416
Myalgia	553 (23.4%)	467 (28.0%)	86 (12.2%)	78 (11.7%)	<0.001
Fatigue	952 (40.3%)	719 (43.2%)	233 (33.2%)	224 (33.8%)	<0.001
Headache	191 (8.0%)	138 (8.3%)	53 (7.5%)	50 (7.5%)	0.551
Nausea or vomiting	116 (4.9%)	84 (5.0%)	32 (4.5%)	30 (4.5%)	0.620
Diarrhea	167 (7.0%)	115 (6.9%)	52 (7.4%)	48 (7.2%)	0.659
Abdominal pain	28 (1.1%)	21 (1.2%)	7 (1.0%)	6 (0.9%)	0.589
Dyspnea	403 (17.0%)	312 (18.7%)	91 (13.0%)	70 (10.5%)	0.001
*Outcome*					
ICU	155 (6.5%)	96 (5.7%)	59 (8.4%)	21 (3.1%)	0.017
MV	96 (3.9%)	55 (3.3%)	39 (5.5%)	19 (2.8%)	0.010
Death	59 (2.4%)	31 (1.8%)	28 (4.0%)	20 (3.0%)	0.002
The mean interval (*d*) (IQR)*					
Admission—ICU	4.4 (1–6)	4.6 (1–6)	4.2 (1–6.5)	8.4 (5–10.5)	0.207
Admission—MV	6.1 (2–9)	6.1 (1–10)	6.1 (2–8.25)	9.6 (5–13.5)	0.758
Admission—death	16.1 (9.5–21.5)	16.5 (9.5–24)	15.6 (10–18)	15.9 (11.8–18.3)	0.386
Admission—discharge	15.7 (7–22)	13.3 (5–9)	19.3 (13–25)	19.3 (13–25)	<0.001

*P*-values show statistically significant differences in features between Cohort 1 and Cohort 2. There were statistically significant differences in prognostic features (e.g., age, dyspnea) in Cohort 1 and Cohort 2, but there was no significant difference in these features of positive cases (refers to the adverse group where patients required ICU admission) in the two cohorts (Table S3). Thus, this difference may be due to the discrepancy in the proportion of Hubei cases (Cohort 1, 69.8%; Cohort 2, 80.1%), which have a higher proportion of severe outcomes (6.9%, 8.6%, respectively).

*Data in parentheses show percentage except for the mean interval where we show interquartile range (IQR). *COPD* chronic obstructive lung disease, *ICU* intensive care unit, *MV* mechanical ventilation.

### Comparison of radiomics models with other modalities

**Fig. 2:**
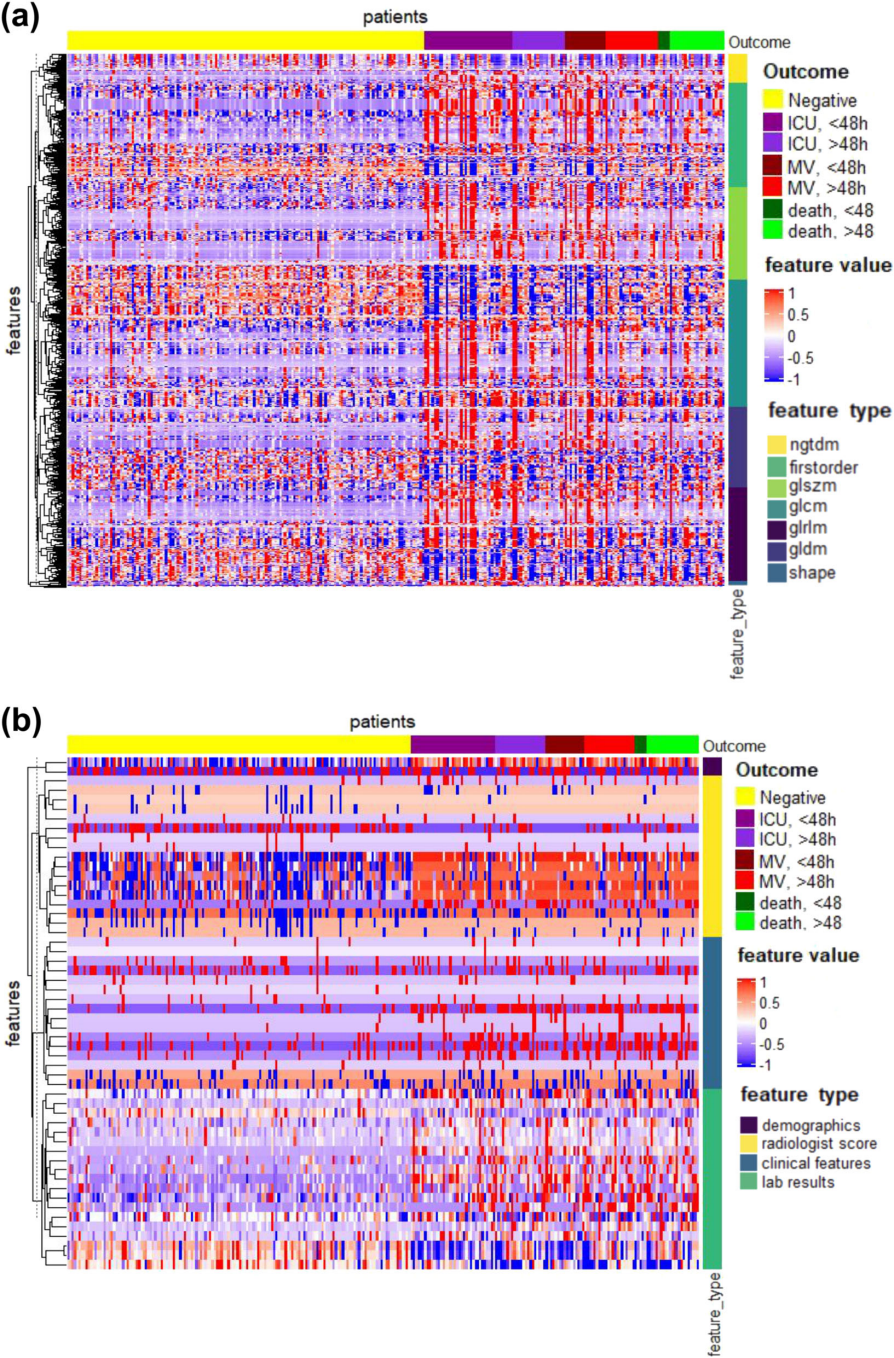
Radiomics and clinical data heatmap. Heatmap showing the prognostic performance of **a** radiomics data and **b** clinical data and R-score data on Cohort 2 with clustering of features. Hundred and fifty negative patients were randomly selected as well as all patients having outcomes of ICU admission, Mechanical Ventilation or Death to draw the heatmap. For patients with more than one adverse outcome, they will appear as samples in each corresponding category. The patients were grouped based on adverse outcomes (i.e., ICU admission, MV, and death) and whether the event occurred within 48 h after admission. The features were clustered within their categories to better visualize the data. The differences between negative outcome patients (yellow) and positive outcome patients can be seen from both (**a**) and (**b**), with some features showing different patterns for negative (patients discharged without any adverse outcomes) or positive patients (patients who required ICU, MV, or death while hospitalized). Almost all CT image features showed good discrimination between negative and severe outcome patients and had more obvious distinctions compared to clinical data. Among clinical data, lab results and demographics had good discriminating power. Part of radiologists’ score features had good discriminating power while clinical features have comparatively weak discriminating power. Regarding the distinctions between ICU admission, mechanical ventilation, and death, CT image features showed better discriminating power than clinical data. In CT image features, from ICU to MV to death, trends of value increasing or decreasing can be observed while in clinical data, this kind of trend is not visible.

**Fig. 3:**
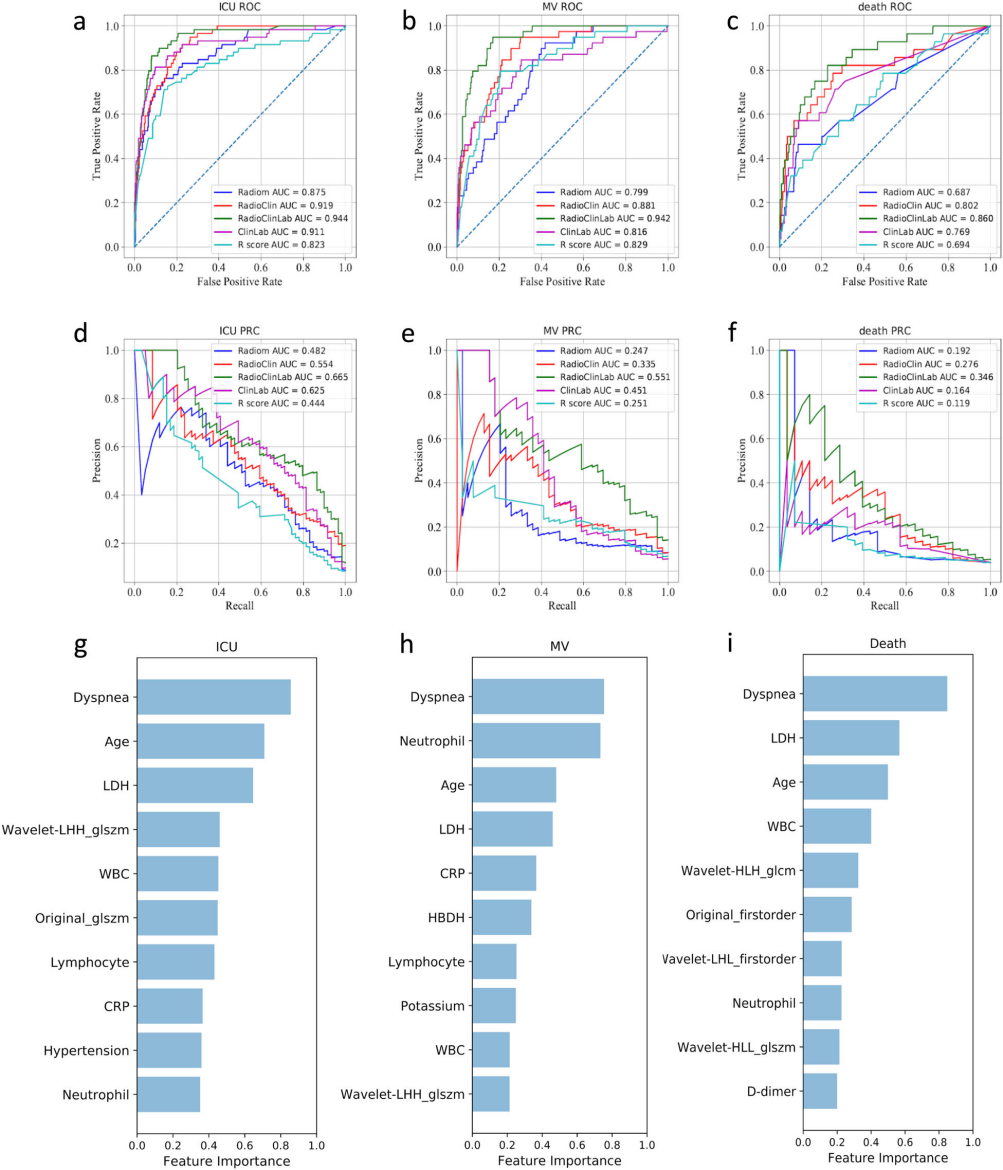
The model performances in the prediction of three outcomes (Cohort 2) and the ten most important features in the three outcome prediction tasks. The first and second row presented ROC curves and PR curves for predicting three events of models based on different data types. **a** and **d**, **b** and **e**, **c** and **f** indicated that RadioClinLab based models for predicting ICU/MV/death achieved the highest AUROC (0.944/0.942/0.860) and AUPRC (0.665/0.551/0.346), respectively. **g**–**i** The ten most important features and their relative importance based on thirty bootstrapping experiments for the three prediction tasks based on the feature importance of the LightGBM classifiers.

We recognized the marked differences of CT-based radiomics data (abbreviated as Radiom), Clinical records (abbreviated as Clin), Laboratory results (abbreviated as Lab), and Radiologists’ semantic data (abbreviated as R-score) on Cohort 1 and Cohort 2 between negative outcome patients (referred to the stable group where patients discharged without any adverse outcome) and positive outcome patients (referred to the adverse group where patients required ICU admission) (Fig. 2, Supplementary Fig. 1, Supplementary Table 2). The optimal models for each data type (i.e., Radiom, RadioClin, RadioClinLab, ClinLab, and R-score) were chosen on Cohort 1 and validated on Cohort 2 and Cohort 3 (Table 2, Supplementary Tables 4 and 5, Fig. 3). On Cohort 2, radiomics features alone (Radiom) showed good performance to predict ICU (AUROC 0.869, AUPRC 0.441), MV (AUROC 0.805, AUPRC 0.245), and death (AUROC 0.667, AUPRC 0.136). When combined with clinical features (RadioClin), the performance of models improved significantly (all three events *p*-value < 0.001) (Supplementary Tables 4 and 5). Notably, as we continued to add the lab results (RadioClinLab), models achieved optimal performance on all three events (AUROC ICU: 0.916, MV: 0.919, death: 0.853; AUPRC ICU: 0.563, MV: 0.476, death: 0.248). RadioClinLab models also outperformed clinical data alone models (ClinLab) (all three events *p*-value < 0.001) (Supplementary Tables 4 and 5, Supplementary Figure 5), suggesting the importance of radiomics features in predicting severe outcomes. Similarly, RadioClinLab models also had good performance on Cohort 3 for ICU (AUROC 0.919, AUPRC 0.348), MV (AUROC 0.943, AUPRC 0.388), and death (AUROC 0.856, AUPRC 0.218). These results demonstrated the models’ ability to predict severe events that occur at least two days after admission (Table 2, Supplementary Table 5).

**Table 2:** Bootstrapping results of the optimal models in Cohort 2 and Cohort 3.

	Cohort 2 (*n* = 700)			Cohort 3 (*n* = 662)		
Data	AUROC (95% CI)	ACC (95% CI)	AUPRC (95% CI)	AUROC (95% CI)	ACC (95% CI)	AUPRC (95% CI)
*ICU*						
Radiom	0.869 (0.857–0.879)	0.864 (0.836–0.889)	0.441 (0.413–0.480)	0.830 (0.809–0.851)	0.876 (0.843–0.907)	0.139 (0.109–0.173)
RadioClin	0.886 (0.854–0.920)	0.917 (0.876–0.936)	0.480 (0.345–0.590)	0.863 (0.825–0.913)	0.954 (0.923–0.971)	0.226 (0.126–0.401)
RadioClinLab	0.916 (0.892–0.945)	0.928 (0.901–0.944)	0.563 (0.397–0.677)	0.919 (0.884–0.962)	0.957 (0.940–0.971)	0.348 (0.192–0.505)
ClinLab	0.860 (0.735–0.924)	0.803 (0.749–0.860)	0.548 (0.348–0.684)	0.906 (0.813–0.971)	0.818 (0.757–0.876)	0.446 (0.294–0.608)
R-score	0.776 (0.725–0.822)	0.916 (0.916–0.917)	0.332 (0.233–0.422)	0.772 (0.722–0.831)	0.968 (0.967–0.968)	0.137 (0.077–0.257)
*MV*						
Radiom	0.805 (0.759–0.844)	0.944 (0.940–0.947)	0.245 (0.178–0.399)	0.760 (0.717–0.831)	0.968 (0.962–0.973)	0.122 (0.089–0.200)
RadioClin	0.869 (0.836–0.912)	0.944 (0.940–0.950)	0.348 (0.282–0.431)	0.867 (0.823–0.917)	0.969 (0.965–0.971)	0.209 (0.161–0.297)
RadioClinLab	0.919 (0.885–0.944)	0.950 (0.944–0.957)	0.476 (0.400–0.616)	0.943 (0.918–0.968)	0.972 (0.967–0.976)	0.388 (0.260–0.533)
ClinLab	0.722 (0.594–0.838)	0.936 (0.927–0.947)	0.312 (0.192–0.450)	0.768 (0.704–0.867)	0.960 (0.949–0.971)	0.303 (0.166–0.477)
R-score	0.804 (0.738–0.854)	0.944 (0.943–0.944)	0.222 (0.171–0.288)	0.736 (0.661–0.841)	0.971 (0.971–0.971)	0.115 (0.074–0.178)
*Death*						
Radiom	0.667 (0.597–0.746)	0.959 (0.954–0.963)	0.136 (0.093–0.194)	0.655 (0.589–0.762)	0.968 (0.964–0.971)	0.104 (0.052–0.178)
RadioClin	0.802 (0.790 0.819)	0.945 (0.937–0.950)	0.281 (0.251–0.315)	0.790 (0.774 0.808)	0.963 (0.957 0.969)	0.286 (0.236–0.345)
RadioClinLab	0.853 (0.799–0.900)	0.960 (0.957–0.963)	0.248 (0.170–0.401)	0.856 (0.804–0.911)	0.969 (0.965–0.973)	0.218 (0.123–0.361)
ClinLab	0.799 (0.758–0.829)	0.938 (0.932–0.945)	0.222 (0.172–0.271)	0.809 (0.761–0.856)	0.956 (0.948–0.963)	0.228 (0.180–0.307)
R-score	0.678 (0.566–0.760)	0.960 (0.960–0.960)	0.120 (0.071–0.206)	0.653 (0.551–0.746)	0.970 (0.968–0.970)	0.092 (0.051–0.249)

*CI* confidence interval, *AUROC* area under the receiver operating characteristics, *AUPRC* area under the precision-recall curve, *ACC* accuracy.

### Comparison of radiomics with radiologists’ scoring

The performance of Radiom models was overall superior to that of radiologist score (R-score) models on two validation cohorts on the three tasks (ICU/MV/death: Cohort 2 AUROC 0.776/0.804/0.678, AUPRC 0.332/0.222/0.120; Cohort 3 AUROC 0.772/0.736/0.653, AUPRC 0.137/0.115/0.092) (Table 2). Specifically, Radiom models had significantly improved predictive value in predicting ICU (*p* < 0.001) and were comparable to R-score models with a higher AUPRC for MV (*p* = 0.003) and death (*p* = 0.021) on Cohort 2. The predictive value of Radiom for ICU and MV happening 2 days later was higher than R-score, while there was no significant difference between these two models on prediction of death on Cohort 3 (Supplementary Tables 5 and 6, Supplementary Fig. 5).

### Key imaging features and clinical prognostic indicators

Among the top-ranking prognostic indicators, clinical data and radiomics features showed a complementary role with no significant correlations (Fig. 3, Supplementary Figs. 6 and 7). In clinical data, mean age >65, dyspnea, higher lactate dehydrogenase (LDH) and inflammatory factors (white blood cell (WBC), neutrophil) are more associated with severe outcomes. Particularly, hypertension and some inflammatory factors (lower lymphocyte, higher C-reactive protein (CRP), and neutrophil)) were valuable for predicting ICU admission, also higher potassium and α-Hydroxybutyrate dehydrogenase (HBDH) and several inflammatory factors (lower lymphocyte, higher CRP) were predictive for MV, while higher D-dimer provided great diagnostic value for death. Most clinical variables were independently correlated with disease progression (Supplementary Note 1). Furthermore, GLSZM-based, GLCM-based, and first-order radiomics features were important features for the prediction of outcomes. In addition, our R-score model suggested that diffuse pulmonary parenchymal ground-glass and consolidative pulmonary opacities in the left upper lobe and pleural effusion increased the adverse outcomes (ICU, MV, death) in COVID-19 patients. Notably, crazy-paving on the initial CT chest was a risk factor of death (Supplementary Table 6, Supplementary Fig. 8).

### Individual severe-event-free survival analysis and performance of time-to-event models

**Fig. 4:**
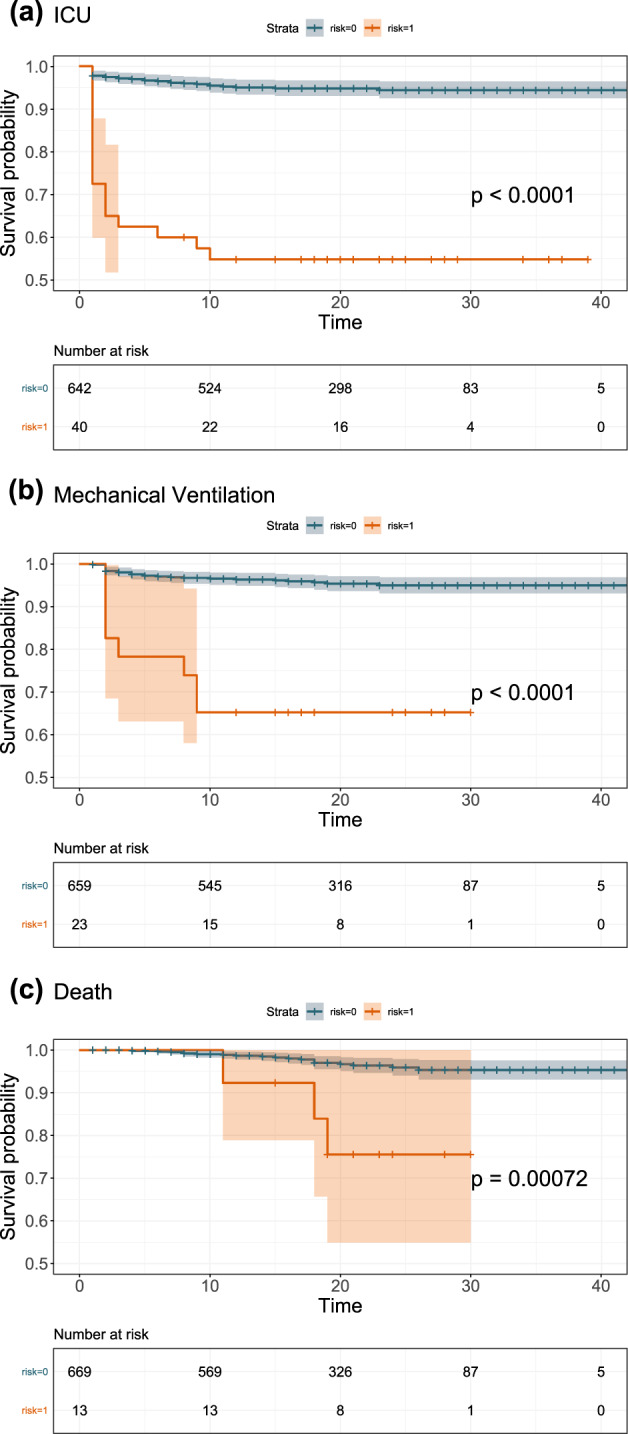
Kaplan–Meier curves for 3 tasks in Cohort 2. Risk groups were divided according to model predicted scores. **a** ICU admission, **b** mechanical ventilation, and **c** death (high-risk: risk = 1, low-risk: risk = 0).

Next, we used time-to-event modeling to stratify survival outcomes of patients. We first separated the patients into high-risk and low-risk groups and evaluated the survival curves of the two groups. Kaplan–Meier curves using the predicted score with the optimal RadioClinLab were generated (Fig. 4). The high-risk group (ICU: 40 observations with 18 events, MV: 23 observations with 8 events, death: 13 observations with 3 events) had a much lower survival probability compared to the low-risk group (ICU: 642 observations with 32 events, MV: 659 observations with 28 events, death: 669 observations with 19 events) in all 3 tasks with a significant statistical difference (*p* < 0.001, log-rank test).

According to the results of time-to-event prediction (Supplementary Table 10) on Cohort 2, the RadioClinLab showed the highest concordance index values on three prediction tasks (0.917, 0.888, and 0.906). In addition, the RadioClinLab outperformed other models on ICU and MV prediction (Brier score 0.061 and 0.053) while the ClinLab model performed best on death prediction (Brier score 0.028). On Cohort 3, RadioClinLab showed the highest concordance index values on three tasks: 0.921, 0.884, and 0.911 and the lowest integrated Brier score on ICU and MV prediction: 0.039 and 0.036 while the ClinLab model showed the lowest integrated Brier score of 0.027. The bootstrapping resampling (Supplementary Table 11) showed that on Cohort 2, RadioClinLab showed the highest concordance index on three tasks (*p* < 0.001, paired one-sided *t*-test) and the lowest integrated Brier score on ICU and MV prediction (*p* < 0.03) while there was no statistically significant difference in the integrated Brier score values between RadioClinLab and ClinLab on death prediction. Generally, these results showed that Radiom, RadioClinLab, and ClinLab models achieved satisfactory performances in time-to-event prediction. In particular, the combination of radiomics features, clinical data, and lab test results contributed most to the prediction and provided the most accurate estimates to the time in days that critical care demands are required.

## Discussion

Our study achieved three goals. First, we provided risk stratification based on CT-based radiomics features and clinical data for COVID-19-infected patients in terms of stable or severe disease (requiring ICU) on admission. Second, our models provided specific outcome prediction (MV and death) for critically ill patients. Finally, we offered insights into estimating time to progression of the severe events (i.e., ICU, MV, and death). This analysis potentially enables rapid stratification and timely intensive care management of patients during this pandemic.

We carefully defined outcome events (i.e., ICU, MV, or death) as prediction labels rather than the general risk severity, so that different medical centers can optimize the resource allocation by utilizing the prediction outcomes. According to our prognosis estimation results, it is possible to request medical resource transfers, such as personnel, local ICU beds, or MV from the Emergency Medical Services command as well as distribution of stable patients from overloaded local ICUs to neighboring affected regions with lower COVID-19 prevalence to balances ICU loads. In addition, the prediction of MV on admission allows for closer monitoring and repetitive assessments of patients over time to determine priority for initiating MV, because there is typically only a limited time window for life saving when the respiratory system deteriorates^14^. Furthermore, combining predictions of demand for medical resources with outcome estimation of death anticipated the need to allocate resources to the patients who are most likely to benefit, which may also help develop priority rationing strategies during pandemics^15^.

Our findings demonstrated the predictive value of CT-based imaging for outcome predictions of CVOID-19 patients. Thin slice chest CT has been an efficient and fast tool for detecting early COVID-19 pneumonia with high sensitivity^16,17^, assessing the disease severity^18–21^, and surveilling the disease progression^22–28^, which provides valuable information to guide clinical management and aid in control of COVID-19^19,29–31^. In our study, the performance of radiomics-based models (Radiom) was better than radiologist’s scores (defined as R-score). Concretely, we found that first-order texture and higher-order radiomics features (i.e., GLSMZ and GLCM-based) were the most important predictors. Our results also indicated that the feature values of diffuse pulmonary parenchymal ground-glass and consolidative pulmonary opacities in the left upper lobe as well as pleural effusion were more associated with the adverse outcomes (ICU, MV, death) in COVID-19 patients, which were consistent with prior findings^32–34^. In addition, crazy-paving was a predictor of death^35^.

Among the identified clinical predictors in our study, age, dyspnea, a liver biochemistry marker (higher lactate dehydrogenase (LDH)) were significant in all three prediction tasks^36–39^. Furthermore, the changes of various inflammatory factors (higher white blood cell (WBC), C-reactive protein (CRP) and neutrophil, and lower lymphocytes) were predictive for the three severe events, consistent with current research that SARS-CoV-2 may accelerate the inflammatory response and cause the fluctuation of inflammatory factors, thereby leading to severe immune injury and lymphopenia^36,37,40–43^. Previous studies also indicated that leukocytosis resulting from a mixed infection of bacteria and fungi in the context of viral pneumonia indicates poor outcomes^44,45^. In addition, our study suggested that electrolyte and acid-base balance (K+) relating to respiratory function and the indicator of myocardial infarction (higher α-Hydroxybutyrate dehydrogenase (HBDH)) contributed to the prediction of progression to severe illness requiring MV, while D-dimer was associated with an increased risk of in-hospital mortality, in agreement with previous studies^11,12,36,42,46^. Other features such as comorbidity (e.g., hypertension) were also related to poor prognosis^37,39^.

Although this study provided insights in using CT-based features to optimize the medical resource allocation based on the patient outcome prediction, our work has several limitations. First, we did not consider the effect of different treatments on the prognosis of patients among clinical centers. In our study, several treatments were adopted including oxygen therapy, MV, ECMO, antiviral treatment, antibiotic treatment, glucocorticoids, and intravenous immunoglobulin therapy. In-depth comparison of different treatment outcomes might improve response prediction. Second, ten well-experienced thoracic radiologists analyzed the CT images in consensus and evaluated tr aditional imaging features in our study, however, we did not study inter-reader variability and such an analysis might need to be addressed in future work. In addition, although our study had a large sample size with clear prognosis information, the numbers of endpoints were limited and only from Chinese hospitals which could potentially limit the generalizability of models in other areas. Finally, additional validation across populations from European and American hospitals is needed to further validate the reported models.

In conclusion, we developed computational models with clinical prognostic estimation functions incorporating CT-based radiomics features as well as clinical data from electronic medical records for COVID-19 patients. This information may aid in delivering proper treatment and optimizing the use of limited medical resources in the current pandemic of COVID-19.

## Methods

### Patient cohort

Our data in this study were collected from 39 hospitals in China (*n* = 3522). Patients selection followed the inclusion criteria: (a) confirmed positive SARS-CoV-2 nucleic acid test; (b) chest CT examinations and laboratory tests on the date of admission; (c) clear short-term prognosis information was available (discharge, or adverse outcomes including the admission to ICU, requiring MV support, and in-hospital death). Along with the exclusion criteria, we collected 2363 patients for analysis (Fig. 1, Supplementary Table 1).

### Data collection and processing

**Fig. 5:**
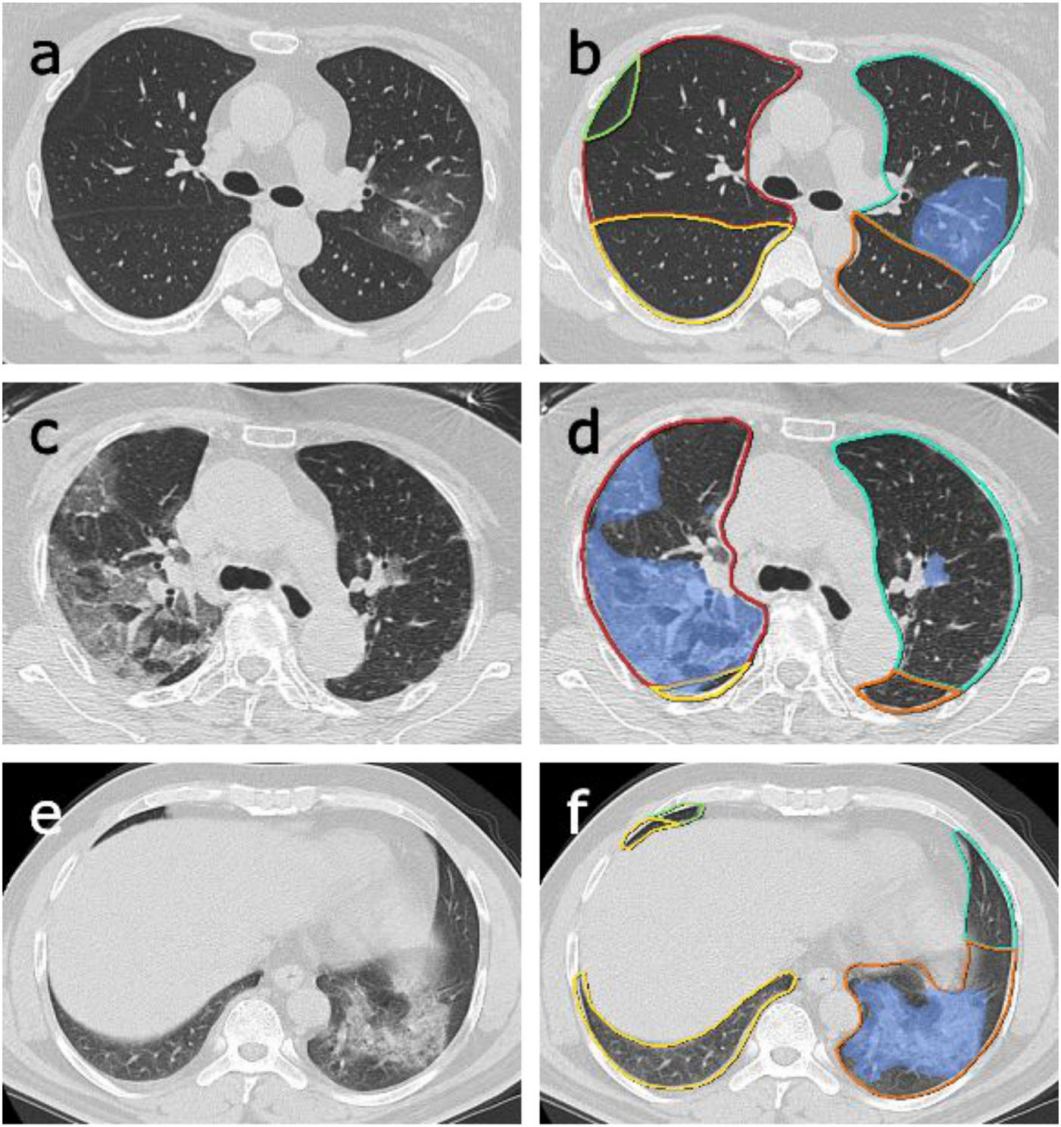
Examples of lesion segmentation by the AI system. Left **a**, **c**, **e**: original images; right **b**, **d**, **f**: pulmonary lobes (colored lines) and opacities segmentation (blue area).

Our multi-modal data (Supplementary Note 2) for each patient included:

(a) Clinical records (abbreviated as Clin): demographics, comorbidities, and clinical symptoms.

(b) Laboratory results (abbreviated as Lab): blood routine, blood biochemistry, coagulation function, infection-related biomarkers. To alleviate missing values that occurred in records, we applied median imputation on the lab data when a missing rate was <50%. Each inpatient received laboratory tests within 24 h after admission and only clinical data on or prior to the date of the CT were used for prediction.

(c) CT-based radiomics features (abbreviated as Radiom): each inpatient underwent a non-contrast chest CT scan within 3 days after admission^47^. A deep-learning AI system (Supplementary Fig. 2, Beijing Deepwise & League of PhD Technology Co. Ltd) was first used to detect and segment the pneumonia lesion (Fig. 5), and two radiologists confirmed the results of the automatic segmentation (average dice = 0.95) (Supplementary Figs. 3, 4). Then pyradiomics (v3.0) running in the Linux platform was adopted to extract radiomic features (1657 features per lesion). Next, for a given patient, for each feature, we summarized the distribution of the feature’s values across all the lesions for the patient by summary statistics (the mean, median, standard deviation, skewness, quartile 1, quartile 3). Finally, a total of 9943 quantitative radiomics features were extracted from CT images for each patient. The radiomics quality score (RQS) of this study is 23 (Supplementary RQS Checklist).

(d) Radiologists’ semantic data (abbreviated as R-score): (1) lesion distribution: subpleural or diffuse; (2) lesion morphology: round or other; (3) main signs: the presence of pure ground-glass opacity (GGO), pure consolidation, GGO with consolidation, interstitial lung disease (ILD), and crazy-paving pattern, (4) other abnormality: pleural effusion; and (5) the total number of lesions and lesion count in each lobe per patient. First, four experienced radiologists annotated 60 randomly selected cases separately as quality assessment, reaching a high intraclass correlation coefficient (0.989, 95% confidence interval 0.983–0.993). Next, they reviewed in consensus on the representative cases to set up the annotation standard. Then, ten radiologists were assigned subsets of CT scans for the annotation task independently without access to the clinical or laboratory results of patients.

(e) Time-to-event data: the three outcome events were defined as the occurrence of the following adverse events through 28 days of follow-up, and they are (a) ICU admission; (b) start to receive MV therapy; and (c) in-hospital death. Discharge criteria and treatment protocols were based on the diagnosis and treatment of novel coronavirus (2019-nCoV) infected pneumonia (trial seventh edition)^29^. The time interval between the dates of admission to the hospital and the corresponding outcomes or discharge were recorded (Supplementary Table 2). Patients’ outcomes were defined as censored if they were transferred to other hospitals during the observation period.

### Feature processing

To address the imbalance and high feature dimensionality in modeling, we adopted several combinations of methods to downsample the negative cases (*n* = 2207, the adverse group where patients required ICU admission) and oversampling the positive cases (*n* = 155, the adverse group where patients required ICU admission, including 94 patients who needed MV and 59 death) to enhance models’ generalizability for the imbalanced data.

Several feature engineering methods were applied: (1) SMOTEENN (synthetic minority oversampling technique and edited nearest neighbors)^48^: The method performs oversampling using SMOTE and cleaning using ENN to deal with imbalanced classes. In this study, a 1:1 (positive cases: negative cases) balanced dataset and a 1:3 imbalanced dataset were created respectively; (2) SMOTEENN + PCA (principal component analysis)^49–51^: upon enlarging the dataset, PCA was applied to reduce the dimensionality of the features. It applies singular value decomposition (SVD) to find the orthogonal principal components and the low-dimension representation of data. In this study, the number of principal components was chosen to explain 0.998 or 0.954 variance; (3) SMOTENN + LASSO feature selection^52^: LASSO feature selection was applied to extract the most important features used in logistic regression with L1 normalization, coefficients of L1 normalization (‘C’) were tuned; (4) SMOTEENN + GUS (generic univariate selection)^53^: Generic univariate selection selects the best features based on univariate statistical tests; (5) SMOTENN + FPR (false-positive rate test)^53^. The feature engineering was done with the toolbox of scikit-learn 0.23.0^53^. In our study, for the last two feature selection methods, F-test and mutual information were used as the scoring function. The feature selection was done with the scikit-learn 0.23.0^53^. The modeling process was done with the raw data and preprocessed data with the methods mentioned above.

### Feature visualization

Feature visualization provides an intuitive manner to understand the distribution of features used in this study. Therefore, we first visualized the distribution of 37 clinical data (including 18 clinical features and 19 laboratory test results), 9943 CT-based radiomics features, and 17 traditional semantic CT features for all patients, with the help of heatmaps and t-distributed Stochastic Neighbor Embedding (t-SNE) in terms of ICU, MV, and death (ComplexHeatmap version 2.2.0)^54,55^. The patients were reasonably grouped based on the adverse outcomes and whether the event occurred within 48 h.

We recognized the marked differences of radiomics data, clinical data, and R-score data on Cohort 1 and Cohort 2 between negative outcome patients and positive outcome patients (Fig. 2, Supplementary Fig. 1). Almost all CT image features showed good discrimination between negative and severe outcome patients and had more obvious distinctions compared to clinical data. Among clinical data, lab results and demographics had good discriminating power. Part of radiologists’ score features had good discriminating power while clinical features have comparatively weak discriminating power. Regarding the distinctions between ICU admission, mechanical ventilation, and death, CT image features showed better discriminating power than clinical data. In CT image features, from ICU to MV to death, trends of value increasing or decreasing can be observed while in clinical data, this kind of trend is not visible.

### Model development and prediction evaluation

There were three binary classification tasks in this study, namely, stable (negative) samples vs. adverse (ICU) samples, non-MV samples vs. MV samples, and survival samples vs. death samples. To test the prediction performances of different data type combinations, multivariable models based on five types of data were developed and compared: (1) radiomics data only (denoted as “Radiom”); (2) radiomics, clinical features (including demographics, comorbidity, and clinical symptoms) (denoted as “RadioClin”); (3) radiomics data, clinical features, and laboratory results data (denoted as “RadioClinLab”); (4) clinical features and laboratory results (denoted as “ClinLab”); (5) radiological score based on the linear combination of semantic imaging features evaluated by radiologists (denoted as “R-score”). To confirm that the patients were reasonably grouped based on the adverse outcomes and whether the event occurred within 48 h, we first provided an intuitive manner to understand the distribution of all types of features used in this study with the help of heatmaps and t-distributed stochastic neighbor embedding (t-SNE) in terms of ICU, MV, and death.

To systematically explore the performance of multiple machine-learning classifiers, we used the following approaches to predict outcomes: (1) Logistic Regression (LR)^56^; (2) Random Forest (RF)^57^; (3) Support Vector Machine (SVM)^58^; (4) Multilayer Perceptron (MLP)^59^; (5) LightGBM^60^. The hyperparameters tuned for each of the algorithms included: (1) LR: the coefficient of L2 normalization (‘C’); (2) RF: the number of estimators (‘n_estimators’), maximum depth (‘max_depth’); (3) SVM: the coefficient of soft margin relaxation (‘C’) with the radial basis function kernel; (4) MLP: the number of hidden units in a two-layer fully connected neural network; (5) LightGBM: learning rate, the number of estimators (‘n_estimators’), the number of leaves (‘num_leaves’). In Cohort 1 (*n* = 1662), the data were split into training and testing sets (ratio 7:3) using stratified random sampling based on death cases. We used fivefold cross-validation on the training set (70% data of Cohort 1) only to tune the model hyperparameters. Both a randomized search with accuracy as the optimization goal and a grid search with F1 score as the optimization goal were implemented on the fivefold cross-validation on the Cohort 1 training set to find the best candidate hyperparameter sets and the predictive performances were evaluated on the test set of Cohort 1 to finalize the hyperparameters associated with each combination of the classifier and the feature engineering method. Finally, to select an optimal model for each prediction task, five models with the top receiver operating characteristic (AUROC)^61^ were firstly selected, and the model with the highest precision-recall (AUPRC)^62^ curves was then chosen as the optimal model for each outcome prediction because AUROC and AUPRC could show model accuracy, precision, and recall in a more comprehensive manner with varying thresholds. Model calibration was performed on the three final RadioClinLab models in the prediction of ICU/MV/death, on Cohort 2 and Cohort 3. The model calibration was based on the Sklearn package in Python via fivefold cross-validation on the training set (Cohort 1) (Supplementary Fig. 9).

### Model external validation and comparison

We tested the statistical difference of the performance of selected models with 30 iterations of bootstrapped resampling on unseen data (Cohort 2 *n* = 700, Cohort 3 *n* = 662, Fig. 1) and used the AUROC and AUPRC curves to estimate their generalization ability. Particularly, with Cohort 3, we could verify models’ ability to predict events that will occur two days later, which may allow the healthcare system to have at least two days to plan ahead and react to the demand for resources. Box plots were also drawn to compare the performances of the optimal models found based on Cohort 1 in three classification tasks. Finally, we selected an optimal model for each prediction task based on the results of the paired one-sided *t*-test, which compared the AUROC and AUPRC of models consisting of different data types (Radiom, RadioClin, RadioClinLab, ClinLab). In addition, we constructed the R-score model using logistic regression based on semantic features to compare with the Radiom model (on both Cohort 2 and Cohort 3) and found out the traditional image features that were helpful to predict the outcome events.

### Analysis of predictive features

We identified the feature importance from the selected optimal models and normalized the highest importance scores in each of the bootstrapping experiments on Cohort 2 (*n* = 700). By taking an average of the feature importance values over 30 bootstrapping experiments, we then focused on the ten most important features for each prediction task. We also plotted the pairplot of the most important features to visualize the relationship of the top ten features. Furthermore, we performed the independent two-sided *t*-test (continuous variables, with normal distribution), proportional z-test (categorical variables), and rank-sum test (continuous variables, without normal distribution) to validate the statistical significance in the feature values of positive cases and all cases in Cohort 1, Cohort 2 after firstly using Shapiro–Wilk normality test.

### Time-to-event modeling

Cox regression with the l_1_ penalty and scikit-survival package 0.12.1 was adopted on time-to-event data in Cohort 1 (*n* = 1277, 77% of the patients originally in Cohort 1 had event time recorded) and Cohort 2 (*n* = 682, 97% of the patients originally in Cohort 2 had event time recorded)^63–66^. Three different data combinations were used for the time-to-event modeling: Radiom, RadioClinLab, and ClinLab. We used fivefold cross-validation on Cohort 1 to determine the “alpha_min_ratio” hyperparameter^65,66^, and calculated the performance on Cohort 2. We used the concordance index (C index) and the integrated Brier score to evaluate the models. On Cohort 1, the optimal model for each data combination was chosen in a similar manner as previously described for the classification tasks by first filtering based on mean C index and then optimizing the mean integrated Brier score on the three tasks. Next, we used Kaplan–Meier analysis to visualize the time-to-event models and the log-rank test to estimate significance. A “high-risk” and “low-risk” group was created according to the predicted score for each patient on each task with the optimal RadioClinLab model. To group the patients into the high-risk group and the low-risk group, we first calculated the ratios of positive cases in Cohort 1, then set thresholds on the predicted probability of the test samples to separate patients according to the ratios based on Cohort 2.

### Statistical analysis

SPSS v15.0 [SPSS Inc., Chicago] and MedCalc statistical software were used for statistical analysis. The Shapiro–Wilk test was used to evaluate the normality of quantitative data among the selected top important features. Mean and standard deviation (SD) were used to describe normally distributed data, while the median and interquartile range (IQR) was used to describe non-normally distributed data. Categorical variables were presented as numbers and percentages. The AUROC, AUPRC, accuracy value, and their 95% CI were listed to assess the model performance. The paired one-sided *t*-test was used to calculate the statistical significance of the difference between each AUROC and AUPRC value in the bootstrapping experiments. Chi-square test and Fisher’s exact test were exploited to compare categorical data while independent *t*-test and Wilcoxon rank-sum test were used to compare the feature values of continuous variables in positive and negative cases in the entire cohort (*n* = 2362). Proportional test was done to compare the feature values of categorical variables in positive and negative cases among the most important features found by classifiers and test the statistical significance of categorical variables between Cohort 1 and Cohort 2. Kaplan–Meier survival analysis was done on the high-risk and low-risk group based on predictions and log-rank test was used to evaluate statistical significance.

### Ethics and registration

The protocol of this multi-center study was approved by the institutional review board of Jinling Hospital, Nanjing University School of Medicine (2020NZKY-005-02). The written informed consent was waived because this was a retrospective study and present no more than minimal risk of harm to subjects and involved no such procedures.

### Reporting summary

Further information on research design is available in the Nature Research Reporting Summary linked to this article.

## Supplementary information

Supplemental Information

Reporting Summary

## Data Availability

The data that support the findings of this study are available on request from the corresponding author (G.M.L.). The data with participant privacy/consent are not publicly available due to hospital regulation restrictions.

## Code availability

The codes that support the findings of this study are available here: https://github.com/terryli710/COVID_19_Rapid_Triage_Risk_Predictor.
