# Supplementary material for: AI-based analysis of CT images for rapid triage of COVID-19 patients
Source: NPJ Digit Med. 2021 Apr 22;4:75. doi: 10.1038/s41746-021-00446-z (PMC8062628; doi:10.1038/s41746-021-00446-z)
Supplement: Supplementary file 1 — Supplemental Information [file 41746_2021_446_MOESM1_ESM.pdf]

Supplementary Note 1 Key imaging features and clinical prognostic indicators

Among the prognostic indicators chosen based on bootstrapping experiments with top-ranking feature importance used in the optimal models for the three prediction tasks (RadioClinLab), clinical data and radiomics features showed a complementary role with no significant correlations (Figure 3, Supplementary Figure 6-7). In clinical data, old age, dyspnea, higher lactate dehydrogenase (LDH) and inflammatory factors (white blood cell (WBC), neutrophil) signaled severe outcomes. Particularly, hypertension and other inflammatory factors (lower lymphocyte, higher C-reactive protein (CRP) and neutrophil)) were valuable for predicting ICU admission, also potassium which related to acid-base and electrolyte disorders, the indicator of myocardial infarction (higher α-Hydroxybutyrate dehydrogenase (HBDH)) and several inflammatory factors (lower lymphocyte, higher CRP) were predictive for MV, while higher D-dimer provided great diagnostic value for death. Furthermore, GLSZM-based, GLCM-based, and first-order radiomics features are important features for the prediction of outcomes. In addition, the top-ranking traditional CT features that contribute most to the R-score model suggested that diffuse pulmonary parenchymal ground-glass and consolidative pulmonary opacities in the left upper lobe and pleural effusion increased the adverse outcomes (ICU, MV, death) in COVID-19 patients. Notably, crazy-paving on the initial CT chest was a risk factor of death. (Supplementary Table 6, Supplementary Figure 7)

The statistical difference between negative and positive cases on the majority of the ten most important features (10/10 for ICU prediction, 10/10 for MV prediction, 9/10 for death prediction) in three outcome prediction tasks on Cohort 1 was confirmed (Supplementary Table 7). On Cohort 2, the feature values of the negative and positive cases on the majority of the ten most important features (10/10 for ICU prediction, 9/10 for MV prediction, 9/10 for death prediction) found by classifiers also showed statistically significant difference (Supplementary Table 8-9). The reason that there were no significant differences in some of the lab results might be that the imputation of the missing values was based on median values on Cohort 1 and the standardization was done based on Cohort 1 (training data). Therefore, due to the distributional differences, the statistical significance was not evident in all the features.

Supplementary Note 2 Data collection

Our multi-modal data included clinical data (clinical records and laboratory results), CT images, and radiologists' semantic features. And the follow-up outcomes with time intervals of each patient.

(1) Clinical records (abbreviated as Clin): (a) demographics: age and gender; (b) comorbidities: coronary heart disease, diabetes, hypertension, chronic obstructive lung disease (COPD), chronic liver disease, chronic kidney disease, and carcinoma; and (c) clinical symptoms: fever, cough, myalgia, fatigue, headache, nausea or vomiting, diarrhea, abdominal pain, and dyspnea.

(2) Laboratory results (abbreviated as Lab): (a) blood routine: white blood cell (WBC) count ($\times 10^9$/L), neutrophil count ($\times 10^9$/L), lymphocyte count ($\times 10^9$/L), platelet count ($\times 10^9$/L), and hemoglobin (g/L); (b) coagulation function: prothrombin time (PT) (s), activated partial thromboplastin time (aPTT) (s), and D-dimer (mg/L); (c) blood biochemistry: albumin (g/L), alanine aminotransferase (ALT) (U/L), aspartate Aminotransferase (AST) (U/L), total bilirubin (mmol/L), serum potassium (mmol/L), sodium (mmol/L), creatinine (μmol/L), creatine kinase (CK) (U/L), lactate dehydrogenase (LDH) (U/L), α-Hydroxybutyrate dehydrogenase (HBDH) (U/L); (d) infection-related biomarkers: C-reactive protein (CRP) (mg/L).

For the laboratory data, we applied median imputation on the lab data when a missing rate was more than 50% to alleviate missing values that occurred in records (Supplementary Table 2). We also performed the sensitivity analysis by changing the imputation approach and testing the performance metrics (AUROC, AUPRC, accuracy). We performed the sensitivity analysis on the ultimate RadioClinLab models based on two protocols on Cohort 2 and Cohort 3: 1) we firstly fixed the median imputation as the imputation of the missing data of the lab test results in the modeling process and imputed the test data based on the minimum, 25 percentile, 75 percentile and maximum values of training data; 2) then, we imputed the training data with the minimum, 25 percentile, 75 percentile and maximum values of the training data and imputed the test data in the same way. According to the results, the model performance has been relatively stable of the imputation approach. There has been a decrease in AUROC and AUPRC when we used extreme values to impute the missing data but the general performances of the model are relatively not very sensitive to the imputation approach (Supplementary Table 3).

(3) CT-based radiomics features (abbreviated as Radiom): a total of 9943 quantitative radiomics features were extracted from CT images for each patient. Pyradiomics (v3.0) running in the Linux platform was adopted to extract radiomic features. Specifically, the radiomics features consisted of lesion count and 6 types of summary statistics of 1,657 original radiomics features extracted from each lesion. The original radiomics features comprised 19 First Order Statistics, 16 Shape-based (3D), 22 Gray Level Cooccurrence Matrix, 16 Gray Level Run Length Matrix, 16 Gray Level Size Zone Matrix, 14 Gray Level Dependence Matrix, 5 Neighboring Gray Tone Difference Matrix features, while the image type consisted of Original, Wavelet, LoG, Square, Logarithm, Gradient and LocalBinaryPattern3D. Then we extracted statistics (mean, standard deviation, median, skewness, quartile 1, quartile 3 of the lesion features, and the lesion count) to associate different lesions with related patients. Finally, a total of 9943 quantitative radiomics features were extracted from CT images for each patient.

The pneumonia was detected and segmented by a deep-learning AI system (Supplementary Figure 2, Beijing Deepwise & League of PhD Technology Co.Ltd), which was built on top of deep convolutional neural networks and proved the performance by previous studies of COVID-19.[1, 2, 3] Three major modules were designed to ensure the final accuracy of this system, i.e., pneumonia lesion detection, pneumonia lesion segmentation and lung lobe segmentation. First, an MVP-Net[4] inspired method was used to detect bounding boxes of pneumonia findings. Channel-wise attention mechanism and multiple inputs (different windows centers and windows widths) were applied to explore the spatial context of pneumonia in order to promise the detect sensitivity, and multiple symptom classifiers were trained to discriminate consolidation, ground-glass opacity, nodules, and so forth. Pneumonia lesions (Supplementary Figure 2), i.e., voxels that contained pneumonia, were extracted by a 3D U-Net.[5] Finally, an anatomical prior embedded network was trained to partition the lung into five pulmonary lobes.[6]

Two radiologists confirmed the results of the automatic segmentation (average dice = 0.95) (Supplementary Figure 3, 4). First, the CT images of 100 patients were randomly selected, and these patients involved various outcome groups (ICU n=7; MV n=5; death n=2; discharge n=96). Secondly, the two radiologists (Q.M.X & C.S.Z.) separately segmented these 100 CT images without knowing the clinical information of these patients (Supplementary Figure 3). Then, the deep-learning AI system was used to automatically detect and segment the 100 cases of pneumonia lesions (Supplementary Figure 3). Finally, we used the overlap of the two radiologists as ground truth and calculated the dice coefficient (average dice = 0.95, Supplementary Figure 4). Our results showed that the deviation between the automatic segmentation algorithm and the radiologists' results was small, which proved the effectiveness of the automatic segmentation algorithm. Furthermore, in our study, ten experienced radiologists (5 to 18 years of experience) additionally reviewed all the automatic delineation results of CT images involved in this study to ensure the quality of segmentation.

All inpatients took the Chest CT scan within three days after admission. The CT scans were performed using ≥ 16 slice multidetector CT scanners (Aquilion ONE / Aquilion PRIME / BrightSpeed / BrightSpeed S / Brilliance 16 / Brilliance 64 / Discovery CT750 HD / eCT / Fluorospot Compact FD / HiSpeed Dual / iCT 256 / Ingenuity CT / Ingenuity Flex / LightSpeed VCT / LightSpeed 16 / NeuViz 16 Classic / Optima CT520 Series / Optima CT540 / Optima CT680 Series / ScintCare CT 16E / Sensation 64 / SOMATOM Definition AS+ / SOMATOM Definition Flash / uCT 510) without use of iodinated contrast agents. To minimize motion artifacts, patients were asked to hold their breath, then axial CT images were acquired during end-inspiration. The CT scan protocols were as follows: tube voltage, 100-120 kVp; effective tube current, 110-250 mAs; detector collimation, 16-320 x 0.625-2.5 mm; slice thickness, 0.625-2.5 mm; pitch, 0.8-1.375. Based on the raw data, the CT images were reconstructed by the iterative reconstruction technique reference needed. To normalize the imaging data collected from multi-center image cohorts, we have normalized all CT spacing to 1.0mm and the pixel spacing in the XY direction to 0.6mm prior to the presentation of the histological characteristics.

(4) Radiologists' semantic data (abbreviated as R-score): qualitative CT image features were evaluated by the ten experienced radiologists (5 to 18 years of experience in thoracic radiology) in the core lab of Jinling Hospital, Medical School of Nanjing University. They independently assessed the axial CT images and/or multiplanar reconstructed images without access to the clinical or laboratory results of patients. Our semantic features of chest thin-slice CT image included (a) lesion distribution: subpleural or diffuse; (b) lesion morphology: round or other; (c) main signs: the presence of pure ground-glass opacity (GGO), pure consolidation, GGO with consolidation, interstitial lung disease (ILD), and crazy-paving pattern, (d) other abnormality: pleural effusion; and (e) the total number of lesions and lesion count in each lobe per patient.

GGO is defined as a fuzzy increase in lung attenuation without obscuring the underlying blood vessels.[7] Consolidation is defined as increased attenuation of the lung parenchyma, blurring the edges of blood vessels and airway walls.[8] ILD is defined as some sparing of individual lobules, forming a geographic-like appearance under the background of GGO, or the distortion of the lung structure and reticular opacities.[8] Crazy-paving pattern is defined as thickened interlobular septa and intralobular lines on the background of ground glass turbidity.[8] Subpleural distribution is defined as the lesion involving the peripheral 1/3 of the lung, while diffuse distribution is defined as continuous involvement without respect to lung segments.[7, 9]

Lungs are classified into five lung lobes. The left lung is divided into upper and lower lobes by oblique fissure, while the right lung is divided into upper, middle, and lower lobes by horizontal fissure and oblique fissure. We counted and recorded the number of lesions in each lobe: 0: no lesion; 1-5: with lesion and the number referred to the lesion number, regardless of the degree and range of the lesions (the highest number is 5). Consensus agreement was achieved through repeated examinations among radiologists.

(5) Time-to-event data: we gathered the following follow-up data. The time intervals between the date of admission and (a) the date of development of adverse outcomes (requiring ICU, MV, and death), or (b) the date of discharge (defined as a patient who had no fever for at least 3 days, a significant improvement on chest CT in both lungs, clinical relief of respiratory symptoms, and repeated negative RT-PCR results at ≥ 24 hours interval) were recorded.

References

1. Ni, Q., et al. A deep learning approach to characterize 2019 coronavirus disease (COVID-19) pneumonia in chest CT images. *Eur Radiol.* 1-11 (2020).
2. Yu, Q., et al. Multicenter cohort study demonstrates more consolidation in upper lungs on initial CT increases the risk of adverse clinical outcome in COVID-19 patients. *Theranostics.* 10:5641-5648 (2020).
3. Wang, Y.C., et al. Dynamic evolution of COVID-19 on chest computed tomography: experience from Jiangsu Province of China. *Eur Radiol.* (2020). https://doi.org/10.1007/s00330-020-06976-6.
4. Li, Z.H., Zhang, S., Zhang, J., Huang, K.Q., Wang, Y.Z. & Yu, Y.Z. MVP-Net: Multi-view FPN with Position-aware Attention for Deep Universal Lesion Detection. *International Conference on Medical Image Computing and Computer-Assisted Intervention*. arXiv:1909.04247 (2019).
5. Ronneberger, O. Fischer, P. & Brox, T. U-net: Convolutional networks for biomedical image segmentation. *In International Conference on Medical Image Computing and Computer-Assisted Intervention*. pages 234–241 (2015).
6. Wang, X.Q., Zhang, Q.Y., Zhou, Z., Yu, Y.Z. & Wang, Y.Z. Evaluating Multi-class Segmentation Errors with Anatomical Prior. *IEEE International Symposium on Biomedical Imaging* (2020).
7. Song, F. et al. Emerging coronavirus 2019-nCoV pneumonia. Radiology. 295: 210-7 (2020).
8. Hansell, D.M., Bankier, A.A., MacMahon, H., McLoud, T.C., Muller, N.L. & Remy, J. Fleischner Society: glossary of terms for thoracic imaging. *Radiology.* 246:697-722 (2008).
9. Ooi, G.C. et al. Severe acute respiratory syndrome: temporal lung changes at thin-section CT in 30 patients. *Radiology*. 230:836-44 (2004).

Supplementary Table 1. Geographical distribution of COVID-19 patients in this study

Province	Total (n = 2362)	Cohort 1			Cohort 2		
		Total (n = 1662)	Stable group	Adverse (ICU) group	Total (n = 700)	Stable group	Adverse (ICU) group
Hubei (HB)	1721	1160	1080	80	561	513	48
Wuhan	830	830	782	48	0	0	0
Huangshi	137	137	123	14	0	0	0
Huanggang	123	123	110	13	0	0	0
Jingzhou	70	70	65	5	0	0	0
Xiaogan	422	0	0	0	422	389	33
Yichang	81	0	0	0	81	72	9
Xiangyang	58	0	0	0	58	52	6
Anhui (AH)	196	196	190	6	0	0	0
Hainan (HI)	129	129	124	5	0	0	0
Chongqing (CQ)	92	92	91	1	0	0	0
Zhejiang (ZJ)	44	44	44	0	0	0	0
Jiangsu (JS)	38	38	38	0	0	0	0
Jiangxi (JX)	51	0	0	0	51	51	0
Hunan (HN)	28	0	0	0	28	27	1
Heilongjiang (HL)	26	0	0	0	26	20	6
Shandong (SD)	21	0	0	0	21	20	1
Henan (HA)	13	0	0	0	13	13	0
Xinjiang (XJ)	1	1	1	0	0	0	0
Guizhou (GZ)	1	1	0	1	0	0	0
Liaoning (LN)	1	1	1	0	0	0	0

Note. The stable group refers to patients who discharged without any adverse outcome; the adverse group includes patients who developed adverse clinical outcomes and were admitted to the ICU (including patients who required mechanical ventilation and those who died).

Supplementary Table 2. Characteristics of patients in stable/adverse (ICU) groups, non-MV/MV groups, and discharge/death groups

	Stable group (n = 2207)	Adverse (ICU) group (n = 155)	*P* value	Non-MV group (n = 2268)	MV group (n = 94)	*P* value	Discharge group (n = 2303)	Death group (n = 59)	*P* value	Missing value (%)
**Clinical feature**										
**Demographics**										
Age (years)	50.802 ± 15.334	64.781 ± 14.179	<.001	51.150 ± 15.501	65.590 ± 12.476	<.001	51.320 ± 15.467	67.390 ± 14.643	<.001	NA
Gender (male)	1138 (51.5%)	91 (58.7%)	.085	1176 (51.8%)	53 (56.3%)	.389	1191 (51.7%)	38 (64.4%)	.054	NA
**Comorbidity**										
Coronary heart disease	135 (6.1%)	37 (23.8%)	<.001	151 (6.6%)	21 (22.3%)	<.001	152 (6.6%)	20 (33.8%)	<.001	NA
Chronic liver disease	81 (3.6%)	1 (0.6%)	.047	81 (3.5%)	1 (1.0%)	.257	81 (3.5%)	1 (1.6%)	.721	NA
Chronic kidney disease	20 (0.9%)	9 (5.8%)	<.001	24 (1.0%)	5 (5.3%)	.005	25 (1.0%)	4 (6.7%)	.005	NA
COPD	36 (1.6%)	15 (9.6%)	<.001	41 (1.8%)	10 (10.6%)	<.001	44 (1.9%)	7 (11.8%)	<.001	NA
Diabetes	224 (10.1%)	37 (23.8%)	<.001	240 (10.5%)	21 (22.3%)	<.001	245 (10.6%)	16 (27.1%)	<.001	NA
Hypertension	427 (19.3%)	73 (47.0%)	<.001	455 (20.0%)	45 (47.8%)	<.001	468 (20.3%)	32 (54.2%)	<.001	NA
Carcinoma	48 (2.1%)	13 (8.3%)	<.001	51 (2.2%)	10 (10.6%)	<.001	55 (2.3%)	6 (10.1%)	.004	NA
**Clinical symptom**										
Fever	1825 (82.6%)	125 (80.6%)	.105	1873 (82.5%)	77 (81.9%)	.274	1906 (82.7%)	44 (74.5%)	.473	NA
Cough	1540 (69.7%)	111 (71.6%)	.529	1579 (69.6%)	72 (76.5%)	.259	1608 (69.8%)	43 (72.8%)	.891	NA
Myalgia	517 (23.4%)	36 (23.2%)	.163	529 (23.3%)	24 (25.5%)	.148	543 (23.5%)	10 (16.9%)	.039	NA
Fatigue	887 (40.1%)	65 (41.9%)	.130	910 (40.1%)	42 (46.6%)	.111	930 (40.3%)	22 (37.2%)	.002	NA
Headache	179 (8.1%)	12 (7.7%)	.022	183 (8.0%)	8 (8.5%)	.084	184 (7.9%)	7 (11.8%)	.023	NA
Nausea or vomiting	104 (4.7%)	12 (7.7%)	.093	111 (4.8%)	5 (5.3%)	.319	109 (4.7%)	7 (11.8%)	.522	NA
Diarrhea	154 (6.9%)	13 (8.3%)	.085	158 (6.9%)	9 (9.5%)	.530	165 (7.1%)	3 (3.3%)	.183	NA

Abdominal pain	25 (1.1%)	3 (1.9%)	.470	27 (1.1%)	1 (1.0%)	.716	28 (1.2%)	0 (0.0%)	.409	NA
Dyspnea	322 (14.5%)	125 (80.6%)	.728	346 (15.2%)	57 (60.6%)	.303	369 (16.0%)	34 (57.6%)	.353	NA
**Laboratory result**										
**Blood routine - no. (%)**										
WBC			<.001						<.001	310 (13.7%)
Increased	97 (97 / 1893)	37 (37 / 144)		106 (106 / 1945)	28 (28 / 92)	<.001	110 (110 / 1980)	24 (24 / 57)		
Decreased	506 (506 / 1893)	19 (19 / 144)		514 (514 / 1945)	11 (11 / 92)		520 (520 / 1980)	5 (5 / 57)		
Neutrophil			<.001			<.001			<.001	455 (20.1%)
Increased	126 (126 / 1745)	52 (52 / 140)		140 (140 / 1794)	38 (38 / 91)		147 (147 / 1828)	31 (31 / 57)		
Decreased	300 (300 / 1745)	9 (9 / 140)		306 (306 / 1794)	3 (3 / 91)		307 (307 / 1828)	2 (2 / 57)		
Lymphocyte			.698			.376			.608	402 (17.8%)
Increased	10 (10 / 1801)	1 (1 / 140)		11 (11 / 1850)	38 (38 / 91)		10 (10 / 1885)	1 (1 / 56)		
Decreased	355 (355 / 1801)	80 (80 / 140)		382 (382 / 1850)	53 (53 / 91)		400 (400 / 1885)	35 (35 / 56)		
Hemoglobin			<.001			.005			.170	432 (19.1%)
Increased	140 (140 / 1770)	5 (5 / 139)		141 (141 / 1822)	4 (4 / 87)		141 (141 / 1856)	4 (4 / 53)		
Decreased	251 (251 / 1770)	58 (58 / 139)		276 (276 / 1822)	33 (33 / 87)		291 (291 / 1856)	18 (18 / 53)		
Platelet			<.001			.007			.109	441 (19.5%)
Increased	83 (83 / 1763)	3 (3 / 138)		94 (94 / 1814)	2 (2 / 87)		94 (94 / 1848)	2 (2 / 53)		
Decreased	93 (93 / 1763)	27 (27 / 138)		96 (96 / 1814)	14 (14 / 87)		102 (102 / 1848)	8 (8 / 53)		
**Coagulation function**										
PT			<.001			.001			.038	853 (37.7%)
Increased	414 (414 / 1344)	58 (58 / 124)		432 (432 / 1389)	40 (40 / 79)		448 (448 / 1422)	24 (24 / 46)		
Decreased	165 (165 / 1344)	6 (6 /124)		169 (169 / 1389)	2 (2 / 79)		169 (169 / 1422)	2 (2 / 46)		

aPTT				<.001			.421			.523	934 (41.3%)
	Increased	240 (240 / 1273)	27 (27 / 112)		248 (248 / 1314)	19 (19 / 71)		254 (254 / 1346)	13 (13 / 39)		
	Decreased	48 (48 / 1273)	11 (11 / 112)		53 (53 / 1314)	6 (6 / 71)		55 (55 / 1346)	4 (4 / 39)		
D dimer											974 (43.1%)
	Increased	735 (735 / 1231)	98 (98 / 111)	<.001	768 (768 / 1270)	65 (65 / 72)	<.001	798 (798 / 1304)	35 (35 / 38)	<.001	
**Infection-related biomarker**											
CRP				<.001			<.001			<.001	461 (20.4%)
	Increased	993 (993 / 1749)	102 (102 / 129)		1028 (1028 / 1796)	67 (67 / 82)		1054 (1054 / 1829)	41 (41 / 49)		
**Blood biochemistry**											
Albumin				<.001			<.001			.103	547 (24.2%)
	Increased	59 (59 / 1653)	1 (1 / 136)		59 (59 / 1703)	1 (1 / 86)		59 (59 / 1740)	1 (1 / 49)		
	Decreased	345 (345 / 1653)	70 (70 / 136)		369 (369 / 1703)	46 (46 / 86)		383 (383 / 1740)	32 (32 / 49)		
ALT				<.001			.001			.165	613 (27.1%)
	Increased	406 (406 / 1596)	50 (50 / 123)		425 (425 / 1641)	31 (31 / 78)		440 (440 / 1675)	16 (16 / 44)		
AST				.005			.120			.040	792 (35.0%)
	Increased	215 (215 / 1406)	43 (43 / 126)		229 (229 / 1449)	29 (29 / 83)		236 (236 / 1484)	22 (22 / 48)		
	Decreased	144 (144 / 1406)	11 (11 / 126)		145 (145 / 1449)	10 (10 / 83)		150 (150 / 1484)	5 (5 / 48)		
Total bilirubin				.139			.235			.610	685 (30.3%)
	Increased	209 (209 / 1527)	42 (42 / 116)		222 (222 / 1568)	29 (29 / 75)		232 (232 / 1601)	19 (19 / 42)		
	Decreased	15 (15 / 1527)	0 (0 / 116)		15 (15 / 1568)	0 (0 / 75)		15 (15 / 1601)	0 (0 / 42)		
Serum potassium				.520			.251			.642	642 (28.4%)
	Increased	113 (113 / 1558)	13 (13 / 132)		118 (118 / 1605)	8 (8 / 85)		120 (120 / 1639)	6 (6 / 51)		

	Decreased	208 (208 / 1558)	30 (30 / 132)		214 (214 / 1605)	24 (24 / 85)		223 (223 / 1639)	15 (15 / 51)		
Sodium				.001			.018			.036	692 (30.6%)
	Increased	60 (60 / 1505)	30 (30 / 132)		70 (70 / 1551)	20 (20 / 86)		76 (76 / 1587)	14 (14 / 50)		
	Decreased	160 (160 / 1505)	31 (31 / 132)		170 (170 / 1551)	21 (21 / 86)		177 (177/ 1587)	14 (14 / 50)		
Creatinine				<.001			.009			<.001	615 (27.2%)
	Increased	69 (69 / 1589)	31 (31 / 129)		82 (82 / 1634)	18 (18 / 84)		78 (78 / 1668)	22 (22 / 50)		
	Decreased	220 (220 / 1589)	29 (29 / 129)		229 (229 / 1634)	20 (20 / 84)		242 (242 / 1668)	7 (7 / 50)		
CK				.034			.014			<.001	765 (33.8%)
	Increased	331 (331 / 1441)	44 (44 / 121)		343 (343 / 1486)	32 (32 / 76)		350 (350 / 1520)	25 (25 / 42)		
	Decreased	638 (638 / 1441)	53 (53 / 121)		659 (659 / 1486)	32 (32 / 76)		683 (683 / 1520)	8 (8 / 42)		
LDH				<.001			<.001			<.001	792 (35.0%)
	Increased	480 (1416)	95 (95 / 117)		512 (512 / 1455)	63 (63 / 78)		538 (538 / 1491)	37 (37 / 42)		
HBDH				.262			.397			.084	1095 (48.4%)
	Increased	488 (488 / 1132)	67 (67 / 87)		505 (505 / 1157)	50 (50 / 62)		524 (524 / 1180)	31 (31 / 39)		
	Decreased	14 (14 / 1132)	4 (4 / 87)		15 (15 / 1157)	3 (3 / 62)		15 (15 / 1180)	3 (3 / 39)		

Note. The stable group refers to patients who discharged without any adverse outcome; the adverse group includes patients who developed adverse clinical outcomes and were admitted to the ICU (including patients required mechanical ventilation and those who died). p value is statistics of comparison between stable and adverse groups. The normal range refers to the criteria of each hospital. Increased means over the upper limit of the normal range and decreased means below the lower limit of the normal range. ± indicates mean ± standard deviation. Data in parentheses show percentage. NA = Not Applicable; COPD = Chronic obstructive lung disease; WBC = White blood cell; PT = Prothrombin time; aPTT = Activated partial thromboplastin time; CRP = C-reactive protein; ALT = Alanine aminotransferase; AST = Aspartate aminotransferase; CK = Creatine kinase; LDH = Lactate dehydrogenase; HBDH = α-Hydroxybutyrate dehydrogenase.

Supplementary Table 3. Sensitivity analysis of the median imputation of the missing values in the lab test results.

					Cohort 2					
RadioClinLab			ICU			MV			Death	
Modeling Imputation	Test Imputation	AUROC	Accuracy	AUPRC	AUROC	Accuracy	AUPRC	AUROC	Accuracy	AUPRC
Median	Median	0.944	0.924	0.665	0.942	0.949	0.551	0.860	0.963	0.346
Median	Minimum	0.940	0.914	0.656	0.928	0.950	0.503	0.760	0.964	0.290
Median	25 percentile	0.938	0.910	0.642	0.925	0.949	0.489	0.860	0.964	0.313
Median	75 percentile	0.935	0.907	0.640	0.916	0.949	0.495	0.859	0.964	0.314
Median	Maximum	0.916	0.884	0.581	0.890	0.936	0.450	0.608	0.963	0.149
Minimum	Minimum	0.910	0.876	0.482	0.910	0.944	0.425	0.854	0.961	0.328
25 percentile	25 percentile	0.898	0.863	0.433	0.927	0.953	0.520	0.859	0.960	0.270
75 percentile	75 percentile	0.906	0.893	0.442	0.936	0.950	0.495	0.854	0.960	0.271
Maximum	Maximum	0.899	0.888	0.418	0.902	0.941	0.314	0.850	0.963	0.281
					Cohort 3					
RadioClinLab			ICU			MV			Death	
Modeling Imputation	Test Imputation	AUROC	Accuracy	AUPRC	AUROC	Accuracy	AUPRC	AUROC	Accuracy	AUPRC
Median	Median	0.948	0.932	0.471	0.955	0.967	0.425	0.882	0.973	0.337
Median	Minimum	0.909	0.924	0.369	0.960	0.971	0.431	0.781	0.973	0.303
Median	25 percentile	0.909	0.920	0.362	0.957	0.970	0.414	0.880	0.973	0.332
Median	75 percentile	0.901	0.915	0.365	0.949	0.967	0.419	0.876	0.973	0.338
Median	Maximum	0.868	0.887	0.282	0.932	0.955	0.367	0.616	0.971	0.101
Minimum	Minimum	0.876	0.875	0.190	0.934	0.968	0.406	0.862	0.970	0.326
25 percentile	25 percentile	0.879	0.879	0.183	0.951	0.967	0.383	0.868	0.970	0.276
75 percentile	75 percentile	0.886	0.897	0.137	0.966	0.971	0.449	0.862	0.970	0.291
Maximum	Maximum	0.893	0.900	0.161	0.927	0.961	0.265	0.841	0.971	0.202

Supplementary Table 4. Performance and algorithms of the optimal models of each data type for the prediction of ICU, MV, and death on the three cohorts

					**ICU**						
			**Cohort 1 (n=1662)**			**Cohort 2 (n = 700)**			**Cohort 3 (n = 662)**		
**Data**	**Feature Engineering**	**Model**	**AUROC**	**ACC**	**AUPRC**	**AUROC**	**ACC**	**AUPRC**	**AUROC**	**ACC**	**AUPRC**
Radiom	SMOTEENN, LASSO C=50	MLP	0.732	0.780	0.261	0.875	0.901	0.482	0.853	0.914	0.183
RadioClin	SMOTEENN, LASSO C=0.5	LightGBM	0.836	0.826	0.383	0.919	0.923	0.554	0.919	0.946	0.332
RadioClinLab	SMOTEENN, LASSO C=1	LightGBM	0.837	0.824	0.307	0.944	0.924	0.665	0.948	0.932	0.471
ClinLab	SMOTEENN, LASSO C=0.2	LR	0.876	0.784	0.335	0.911	0.764	0.626	0.958	0.759	0.539
R-score	/	LR	0.600	0.939	0.096	0.823	0.916	0.444	0.813	0.968	0.167
					**MV**						
			**Cohort 1 (n=1662)**			**Cohort 2 (n = 700)**			**Cohort 3 (n = 662)**		
**Data**	**Feature Engineering**	**Model**	**AUROC**	**ACC**	**AUPRC**	**AUROC**	**ACC**	**AUPRC**	**AUROC**	**ACC**	**AUPRC**
Radiom	SMOTEENN 1:3	LightGBM	0.823	0.954	0.307	0.799	0.946	0.247	0.753	0.968	0.154
RadioClin	SMOTEENN 1:3	LightGBM	0.836	0.826	0.383	0.881	0.946	0.335	0.874	0.967	0.225
RadioClinLab	SMOTEENN, LASSO C=1	LightGBM	0.850	0.970	0.420	0.942	0.949	0.551	0.955	0.967	0.425
ClinLab	SMOTEENN 1:3	MLP	0.876	0.784	0.335	0.816	0.927	0.451	0.814	0.953	0.387
R-score	/	LR	0.607	0.967	0.065	0.829	0.944	0.251	0.670	0.970	0.105
					**Death**						
			**Cohort 1 (n=1662)**			**Cohort 2 (n = 700)**			**Cohort 3 (n = 662)**		
**Data**	**Feature Engineering**	**Model**	**AUROC**	**ACC**	**AUPRC**	**AUROC**	**ACC**	**AUPRC**	**AUROC**	**ACC**	**AUPRC**
Radiom	SMOTEENN, LASSO C=1	RF	0.881	0.970	0.300	0.687	0.960	0.192	0.680	0.968	0.123
RadioClin	SMOTEENN, FPR, F-Classif	LR	0.948	0.960	0.395	0.802	0.950	0.276	0.788	0.965	0.298
RadioClinLab	SMOTEENN, LASSO C=1	SVM	0.826	0.984	0.417	0.860	0.963	0.346	0.882	0.973	0.337
ClinLab	SMOTEENN, LASSO C=30	MLP	0.838	0.968	0.121	0.769	0.941	0.164	0.805	0.964	0.206
R-score	/	LR	0.704	0.979	0.056	0.694	0.960	0.119	0.670	0.970	0.105

Supplementary Table 5. Statistical significance of the bootstrapping results of different data modalities in Cohort 2 and Cohort 3

Model comparison		Cohort 2				Cohort 3			
				**ICU**					
**model 1**	**model 2**	**Z_AUROC**	**p-value**	**Z_AUPRC**	**p-value**	**Z_AUROC**	**p-value**	**Z_AUPRC**	**p-value**
Radiom	R-score	16.624	<0.001	10.089	<0.001	7.950	<0.001	0.866	0.197
RadioClin	Radiom	5.591	<0.001	2.538	0.008	6.545	<0.001	6.147	<0.001
RadioClinLab	RadioClin	7.834	<0.001	5.17	<0.001	7.994	<0.001	4.969	<0.001
RadioClinLab	ClinLab	5.702	<0.001	0.816	0.211	1.244	0.112	-3.834	0.001
				**MV**					
**Model comparison**		**Cohort 2**				**Cohort 3**			
**model 1**	**model 2**	**Z_AUROC**	**p-value**	**Z_AUPRC**	**p-value**	**Z_AUROC**	**p-value**	**Z_AUPRC**	**p-value**
Radiom	R-score	0.254	0.401	2.947	0.003	7.834	<0.001	15.648	<0.001
RadioClin	Radiom	12.652	<0.001	10.023	<0.001	12.652	<0.001	10.023	<0.001
RadioClinLab	RadioClin	9.184	<0.001	8.711	<0.001	15.687	<0.001	2.866	0.004
RadioClinLab	ClinLab	15.186	<0.001	9.229	<0.001	18.132	<0.001	4.218	<0.001
				**Death**					
**Model comparison**		**Cohort 2**				**Cohort 3**			
**model 1**	**model 2**	**Z_AUROC**	**p-value**	**Z_AUPRC**	**p-value**	**Z_AUROC**	**p-value**	**Z_AUPRC**	**p-value**
Radiom	R-score	-0.869	0.146	2.119	0.021	0.161	0.436	1.169	0.126
RadioClin	Radiom	19.518	<0.001	25.452	<0.001	14.206	<0.001	20.231	<0.001
RadioClinLab	RadioClin	11.486	<0.001	-3.654	0.001	9.956	<0.001	-5.646	<0.001
RadioClinLab	ClinLab	9.217	<0.001	2.106	0.022	5.336	<0.001	-0.969	0.170

Supplementary Table 6. The feature weight of traditional CT-based features on three outcome prediction tasks.

Feature	ICU	MV	Death
**Lesion distribution**			
Subpleural	-0.070	-0.114	0.166
Diffuse	0.177	0.290	0.393
**Lesion morphology**			
Round	-0.026	-0.230	-0.367
Other	-0.023	0	-0.086
**Main sign**			
GGO	0.137	0.141	0
Pure consolidation	0.137	0.156	0.055
GGO with consolidation	0.048	0.097	0.113
ILD	-0.057	0	0
Crazy-paving pattern	0	0.098	0.117
**Other abnormality**			
Pleural effusion	0.224	0.205	0.150
**Number of lesions in each lobe**			
RUL	0	0.036	-0.005
RML	0.134	0	0
RLL	0	0	0
LUL	0.357	0.292	0.131
LLL	0.014	0.013	0
**Lesion count**			
Single	-0.007	0	0
Multiple	0	0	-0.227

Note. GGO = the presence of pure ground-glass opacity; ILD = interstitial lung disease ILD; RRL = right upper lobe; RML = right middle lobe; RLL = right lower lobe; LUL = left upper lobe; LLL = left lower lobe

Supplementary Table 7. The statistical significance of the difference between negative and positive cases on all of the ten most important features in three outcome prediction tasks on Cohort 1.

Feature	Outcome	Top 10 Feature	Group 1	Group 2	Z score	p_value
wavelet-LHH_glszm_LargeAreaHighGrayLevelEmphasis_std	ICU	T	ICU1	ICU0	5.710	<0.001
	MV	T	MV1	MV0	4.897	<0.001
	Death	F	Death1	Death0	1.667	0.096
original_glszm_SmallAreaLowGrayLevelEmphasis_std	ICU	T	ICU1	ICU0	-6.751	<0.001
	MV	F	MV1	MV0	-4.622	<0.001
	Death	F	Death1	Death0	-4.267	<0.001
LDH	ICU	T	ICU1	ICU0	7.522	<0.001
	MV	T	MV1	MV0	6.766	<0.001
	Death	T	Death1	Death0	5.335	<0.001
Age	ICU	T	ICU1	ICU0	7.115	<0.001
	MV	T	MV1	MV0	6.348	<0.001
	Death	T	Death1	Death0	4.808	<0.001
WBC	ICU	T	ICU1	ICU0	5.945	<0.001
	MV	T	MV1	MV0	5.866	<0.001
	Death	T	Death1	Death0	3.957	<0.001
Lymphocyte	ICU	T	ICU1	ICU0	-7.456	<0.001
	MV	T	MV1	MV0	-6.549	<0.001
	Death	F	Death1	Death0	-4.181	<0.001
Potassium	ICU	F	ICU1	ICU0	-2.359	0.018
	MV	T	MV1	MV0	-2.842	0.004
	Death	F	Death1	Death0	-1.325	0.185
C-reactive Protein	ICU	T	ICU1	ICU0	6.414	<0.001
	MV	T	MV1	MV0	6.362	<0.001
	Death	F	Death1	Death0	4.691	<0.001
Neutrophil	ICU	T	ICU1	ICU0	7.341	<0.001
	MV	T	MV1	MV0	7.680	<0.001
	Death	T	Death1	Death0	4.418	<0.001
HBDH	ICU	F	ICU1	ICU0	6.008	<0.001
	MV	T	MV1	MV0	5.641	<0.001
	Death	F	Death1	Death0	3.057	0.002

wavelet-HLH_glcm_InverseVariance_75	ICU	F	ICU1	ICU0	-5.085	<0.001
	MV	F	MV1	MV0	-5.156	<0.001
	Death	T	Death1	Death0	-6.302	<0.001
original_firstorder_Minimum_75	ICU	F	ICU1	ICU0	-5.634	<0.001
	MV	F	MV1	MV0	-5.447	<0.001
	Death	T	Death1	Death0	-6.211	<0.001
wavelet-LHL_firstorder_Skewness_medium	ICU	F	ICU1	ICU0	4.555	<0.001
	MV	F	MV1	MV0	3.534	<0.001
	Death	T	Death1	Death0	4.889	<0.001
wavelet-HLL_glszm_LargeAreaLowGrayLevelEmphasis_std	ICU	F	ICU1	ICU0	0.941	0.346
	MV	F	MV1	MV0	-0.584	0.559
	Death	T	Death1	Death0	-0.521	0.602
D-dimer	ICU	F	ICU1	ICU0	5.909	<0.001
	MV	F	MV1	MV0	5.581	<0.001
	Death	T	Death1	Death0	3.249	0.001
Dyspnea	ICU	T	ICU1	ICU0	7.094	<0.001
	MV	T	MV1	MV0	7.572	<0.001
	Death	T	Death1	Death0	5.099	<0.001
Hypertension	ICU	T	ICU1	ICU0	5.117	<0.001
	MV	F	MV1	MV0	3.972	<0.001
	Death	F	Death1	Death0	2.942	0.003

Supplementary Table 8. The statistical significance of the difference between negative and positive cases on all of the ten most important features in three outcome prediction tasks on Cohort 2

Feature	Outcome	Top 10 Feature	Group 1	Group 2	Z score	p_value
wavelet-LHH_glszm_LargeAreaHighGrayLevelEmphasis_std	ICU	T	ICU1	ICU0	5.940	<0.001
	MV	T	MV1	MV0	4.801	<0.001
	Death	F	Death1	Death0	2.825	0.005
original_glszm_SmallAreaLowGrayLevelEmphasis_std	ICU	T	ICU1	ICU0	-4.333	<0.001
	MV	F	MV1	MV0	-2.982	0.003
	Death	F	Death1	Death0	-2.151	0.032
LDH	ICU	T	ICU1	ICU0	8.774	<0.001
	MV	T	MV1	MV0	7.535	<0.001
	Death	T	Death1	Death0	4.795	<0.001
Age	ICU	T	ICU1	ICU0	8.402	<0.001
	MV	T	MV1	MV0	6.523	<0.001
	Death	T	Death1	Death0	5.766	<0.001
WBC	ICU	T	ICU1	ICU0	3.239	0.001
	MV	T	MV1	MV0	3.471	0.001
	Death	T	Death1	Death0	3.828	<0.001
Lymphocyte	ICU	T	ICU1	ICU0	-6.192	<0.001
	MV	T	MV1	MV0	-6.635	<0.001
	Death	F	Death1	Death0	-6.077	<0.001
Potassium	ICU	F	ICU1	ICU0	-0.554	0.580
	MV	T	MV1	MV0	-1.059	0.290
	Death	F	Death1	Death0	-2.160	0.031
C-reactive Protein	ICU	T	ICU1	ICU0	3.193	0.001
	MV	T	MV1	MV0	3.423	0.001
	Death	F	Death1	Death0	2.661	0.008
Neutrophil	ICU	T	ICU1	ICU0	4.877	<0.001

	MV	T	MV1	MV0	5.534	<0.001
	Death	T	Death1	Death0	5.481	<0.001
HBDH	ICU	F	ICU1	ICU0	2.453	0.014
	MV	T	MV1	MV0	3.315	0.001
	Death	F	Death1	Death0	4.490	<0.001
wavelet-HLH_glcm_InverseVariance_75	ICU	F	ICU1	ICU0	-2.586	0.010
	MV	F	MV1	MV0	-2.236	0.025
	Death	T	Death1	Death0	-1.878	0.060
original_firstorder_Minimum_75	ICU	F	ICU1	ICU0	-6.993	<0.001
	MV	F	MV1	MV0	-5.670	<0.001
	Death	T	Death1	Death0	-3.972	<0.001
wavelet-LHL_firstorder_Skewness_medium	ICU	F	ICU1	ICU0	5.687	<0.001
	MV	F	MV1	MV0	3.528	<0.001
	Death	T	Death1	Death0	2.346	0.019
wavelet-HLL_glszm_LargeAreaLowGrayLevelEmphasis_std	ICU	F	ICU1	ICU0	1.051	0.293
	MV	F	MV1	MV0	1.536	0.124
	Death	T	Death1	Death0	2.779	0.005
D-dimer	ICU	F	ICU1	ICU0	8.716	<0.001
	MV	F	MV1	MV0	7.106	<0.001
	Death	T	Death1	Death0	5.154	<0.001
Dyspnea	ICU	T	ICU1	ICU0	11.667	<0.001
	MV	T	MV1	MV0	9.232	<0.001
	Death	T	Death1	Death0	7.626	<0.001
Hypertension	ICU	T	ICU1	ICU0	6.921	<0.001
	MV	F	MV1	MV0	5.427	<0.001
	Death	F	Death1	Death0	6.370	<0.001

Supplementary Table 9. Statistical significance test of top ten important feature values of positive cases between Cohort 1 and Cohort 2

ICU		
**Feature**	**Z_score**	**p_value**
Dyspnea	-1.613	0.107
Age	-1.268	0.207
LDH	-4.122	<0.001
wavelet-LHH_glszm_LargeAreaHighGrayLevelEmphasis_std	-3.206	0.001
WBC	1.732	0.083
original_glszm_SmallAreaLowGrayLevelEmphasis_std	-0.359	0.719
Lymphocyte	-0.945	0.345
C-reactive Protein	-0.258	0.796
Hypertension	-1.021	0.307
Neutrophil	-1.013	0.311
**MV**		
**Feature**	**Z_score**	**p_value**
Dyspnea	-0.042	0.967
Neutrophil	0.837	0.403
Age	0.129	0.898
LDH	-3.017	0.003
C-reactive Protein	0.284	0.776
HBDH	0.269	0.788
Lymphocyte	1.211	0.226
Potassium	-0.214	0.830
WBC	1.152	0.249
wavelet-LHH_glszm_LargeAreaHighGrayLevelEmphasis_std	-2.328	0.020
**Death**		
**Feature**	**Z_score**	**p_value**
Dyspnea	-0.350	0.726
LDH	-0.682	0.495
Age	-0.470	0.640
WBC	0.104	0.917
wavelet-HLH_glcm_InverseVariance_75	-0.696	0.486
original_firstorder_Minimum_75	-4.393	<0.001

wavelet-LHL_firstorder_Skewness_medium	1.793	0.073
Neutrophil	-0.407	0.684
wavelet-HLL_glszm_LargeAreaLowGrayLevelEmphasis_std	-2.126	0.033
D-dimer	-1.252	0.211

Supplementary Table 10. Results of time-to-event prediction with Cox regression models

	Cohort 2 (n = 682)					
	ICU		MV		Death	
Data	C Index	Integrated Brier Score	C Index	Integrated Brier Score	C Index	Integrated Brier Score
Radiom	0.878	0.073	0.850	0.062	0.730	0.052
RadioClinLab	0.917	0.061	0.888	0.053	0.906	0.045
ClinLab	0.857	0.069	0.807	0.063	0.813	0.028
	**Cohort 3 (n = 652)**					
	**ICU**		**MV**		**Death**	
**Data**	**C Index**	**Integrated Brier Score**	**C Index**	**Integrated Brier Score**	**C Index**	**Integrated Brier Score**
Radiom	0.811	0.055	0.771	0.041	0.691	0.043
RadioClinLab	0.921	0.039	0.884	0.036	0.911	0.036
ClinLab	0.896	0.042	0.857	0.046	0.845	0.027

Supplementary Table 11. Bootstrapping results of Cox regression model for time-to-event prediction on Cohort 2 and Cohort 3

Cohort 2 (n = 682) Bootstrapping Results						
	ICU		MV		Death	
Data	C Index (mean, 95%CI)	Integrated Brier Score (mean, 95%CI)	C Index (mean, 95%CI)	Integrated Brier Score (mean, 95%CI)	C Index (mean, 95%CI)	Integrated Brier Score (mean, 95%CI)
Radiom	0.844 (0.795-0.893)	0.095 (0.065-0.160)	0.808 (0.731-0.863)	0.076 (0.055-0.132)	0.709 (0.618-0.792)	0.059 (0.041-0.095)
RadioClinLab	0.893 (0.792-0.935)	0.077 (0.062-0.114)	0.877 (0.824-0.930)	0.066 (0.047-0.129)	0.869 (0.775-0.905)	0.046 (0.035-0.065)
ClinLab	0.822 (0.761-0.876)	0.075 (0.064-0.093)	0.773 (0.679-0.880)	0.071 (0.061-0.091)	0.767 (0.624-0.911)	0.042 (0.030-0.076)

Cohort 3 (n = 652) Bootstrapping Results						
	ICU		MV		Death	
Data	C Index (mean, 95%CI)	Integrated Brier Score (mean, 95%CI)	C Index (mean, 95%CI)	Integrated Brier Score (mean, 95%CI)	C Index (mean, 95%CI)	Integrated Brier Score (mean, 95%CI)
Radiom	0.783 (0.734-0.845)	0.072 (0.039-0.133)	0.728 (0.639-0.808)	0.058 (0.034-0.131)	0.686 (0.591-0.771)	0.046 (0.038-0.064)
RadioClinLab	0.885 (0.840-0.931)	0.061 (0.037-0.134)	0.877 (0.822-0.939)	0.047 (0.030-0.152)	0.870 (0.769-0.929)	0.038 (0.032-0.065)
ClinLab	0.877 (0.813-0.935)	0.046 (0.033-0.070)	0.831 (0.751-0.894)	0.048 (0.036-0.059)	0.790 (0.643-0.908)	0.033 (0.026-0.052)

Table 1. The radiomics quality score (RQS) of this study

Criteria	Points
Image protocol quality - well-documented image protocols (for example, contrast, slice thickness, energy, etc.) and/or usage of public image protocols allow reproducibility/replicability	2
Multiple segmentations - possible actions are: segmentation by different physicians/algorithms/software, perturbing segmentations by (random) noise, segmentation at different breathing cycles. Analyse feature robustness to segmentation variabilities	1
Phantom study on all scanners - detect inter-scanner differences and vendor-dependent features. Analyse feature robustness to these sources of variability	0
Imaging at multiple time points - collect images of individuals at additional time points. Analyse feature robustness to temporal variabilities (for example, organ movement, organ expansion/ shrinkage)	0
Feature reduction or adjustment for multiple testing - decreases the risk of overfitting. Overfitting is inevitable if the number of features exceeds the number of samples. Consider feature robustness when selecting features	3
Multivariable analysis with non radiomics features (for example, EGFR mutation) - is expected to provide a more holistic model. Permits correlating/inferencing between radiomics and non radiomics features	1
Detect and discuss biological correlates - demonstration of phenotypic differences (possibly associated with underlying gene–protein expression patterns) deepens understanding of radiomics and biology	1
Cut-off analyses - determine risk groups by either the median, a previously published cut-off or report a continuous risk variable. Reduces the risk of reporting overly optimistic results	1
Discrimination statistics - report discrimination statistics (for example, C-statistic, ROC curve, AUC) and their statistical significance (for example, p-values, confidence intervals). One can also apply resampling method (for example, bootstrapping, cross-validation)	2
Calibration statistics - report calibration statistics (for example, Calibration-in-the-large/slope, calibration plots) and their statistical significance (for example, P-values, confidence intervals). One can also apply resampling method (for example, bootstrapping, cross-validation)	2
Prospective study registered in a trial database - provides the highest level of evidence supporting the clinical validity and usefulness of the radiomics biomarker	0
Validation - the validation is performed without retraining and without adaptation of the cut-off value, provides crucial information with regard to credible clinical performance	5
Comparison to 'gold standard' - assess the extent to which the model agrees with/is superior to the current 'gold standard' method (for example, TNM-staging for survival prediction). This comparison shows the added value of radiomics	2
Potential clinical utility - report on the current and potential application of the model in a clinical setting (for example, decision curve analysis).	2
Cost-effectiveness analysis - report on the cost-effectiveness of the clinical application (for example, QALYs generated)	0
Open science and data - make code and data publicly available. Open science facilitates knowledge transfer and reproducibility of the study	1
Total points (36 = 100%)	23

TRIPOD Checklist: Prediction Model Development and Validation

Section/Topic	Ite		Checklist Item	Page
**Title and abstract**				
Title	1	D;V	Identify the study as developing and/or validating a multivariable prediction model, the target population, and the outcome to be predicted.	1
Abstract	2	D;V	Provide a summary of objectives, study design, setting, participants, sample size, predictors, outcome, statistical analysis, results, and conclusions.	2
**Introduction**				
Background and objectives	3a	D;V	Explain the medical context (including whether diagnostic or prognostic) and rationale for developing or validating the multivariable prediction model, including references to existing models.	2
	3b	D;V	Specify the objectives, including whether the study describes the development or validation of the model or both.	2
**Methods**				
Source of data	4a	D;V	Describe the study design or source of data (e.g., randomized trial, cohort, or registry data), separately for the development and validation data sets, if applicable.	2,5
	4b	D;V	Specify the key study dates, including start of accrual; end of accrual; and, if applicable, end of follow-up.	2,5,6,S
Participants	5a	D;V	Specify key elements of the study setting (e.g., primary care, secondary care, general population) including number and location of centres.	5,6,7,S
	5b	D;V	Describe eligibility criteria for participants.	5,S
	5c	D;V	Give details of treatments received, if relevant.	6
Outcome	6a	D;V	Clearly define the outcome that is predicted by the prediction model, including how and when assessed.	6
	6b	D;V	Report any actions to blind assessment of the outcome to be predicted.	6
Predictors	7a	D;V	Clearly define all predictors used in developing or validating the multivariable prediction model, including how and when they were measured.	5,6,S
	7b	D;V	Report any actions to blind assessment of predictors for the outcome and other predictors.	6,7
Sample size	8	D;V	Explain how the study size was arrived at.	5
Missing data	9	D;V	Describe how missing data were handled (e.g., complete-case analysis, single imputation, multiple imputation) with details of any imputation method.	5
Statistical analysis methods	10a	D	Describe how predictors were handled in the analyses.	7
	10b	D	Specify type of model, all model-building procedures (including any predictor selection), and method for internal validation.	6
	10c	V	For validation, describe how the predictions were calculated.	7
	10d	D;V	Specify all measures used to assess model performance and, if relevant, to compare multiple models.	6,7,S
	10e	V	Describe any model updating (e.g., recalibration) arising from the validation, if done.	S
Risk groups	11	D;V	Provide details on how risk groups were created, if done.	6
Development vs. validation	12	V	For validation, identify any differences from the development data in setting, eligibility criteria, outcome, and predictors.	5,6,7,S
**Results**				
Participants	13a	D;V	Describe the flow of participants through the study, including the number of participants with and without the outcome and, if applicable, a summary of the follow-up time. A diagram may be helpful.	2,3,S
	13b	D;V	Describe the characteristics of the participants (basic demographics, clinical features, available predictors), including the number of participants with missing data for predictors and outcome.	2,3,S
	13c	V	For validation, show a comparison with the development data of the distribution of important variables (demographics, predictors and outcome).	3,4
Model development	14a	D	Specify the number of participants and outcome events in each analysis.	3
	14b	D	If done, report the unadjusted association between each candidate predictor and outcome.	3
Model specification	15a	D	Present the full prediction model to allow predictions for individuals (i.e., all regression coefficients, and model intercept or baseline survival at a given time point).	3,4
	15b	D	Explain how to the use the prediction model.	3,4
Model performance	16	D;V	Report performance measures (with CIs) for the prediction model.	3,4,S
Model-updating	17	V	If done, report the results from any model updating (i.e., model specification, model performance).	5,6,S
**Discussion**				
Limitations	18	D;V	Discuss any limitations of the study (such as nonrepresentative sample, few events per predictor, missing data).	5
Interpretation	19a	V	For validation, discuss the results with reference to performance in the development data, and any other validation data.	5
	19b	D;V	Give an overall interpretation of the results, considering objectives, limitations, results from similar studies, and other relevant evidence.	4,5
Implications	20	D;V	Discuss the potential clinical use of the model and implications for future research.	5
**Other information**				
Supplementary information	21	D;V	Provide information about the availability of supplementary resources, such as study protocol, Web calculator, and data sets.	3-7,S
Funding	22	D;V	Give the source of funding and the role of the funders for the present study.	8

*Items relevant only to the development of a prediction model are denoted by D, items relating solely to a validation of a prediction model are denoted by V, and items relating to both are denoted D;V. S refers to supplementary materials.

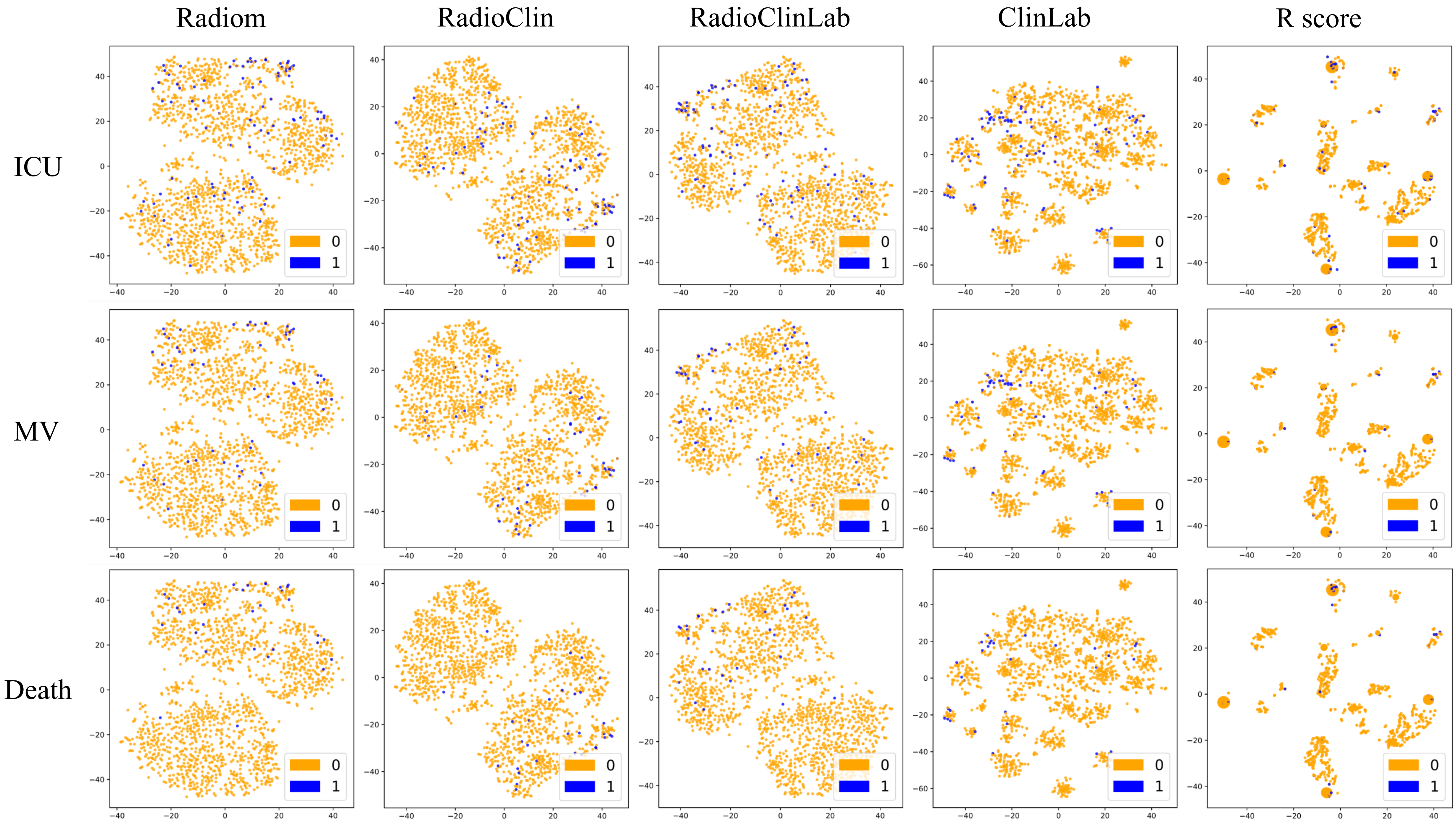

Radiom
RadioClin
RadioClinLab
ClinLab
R score
ICU
MV
Death
0
1

Supplementary Figure 1. t-SNE visualization of cases in Cohort 1 with five different combinations of data types.

Each point denotes a patient, where yellow represents patients discharge without any adverse outcome and blue represents patients with events for ICU, MV, or death. The distance between each point is positively related to the similarity of that lesion of patients. Note: with only the raw data, the positive cases and negative cases are not visually well separated. This calls for necessary feature engineering steps and classifiers to select features important to classification.

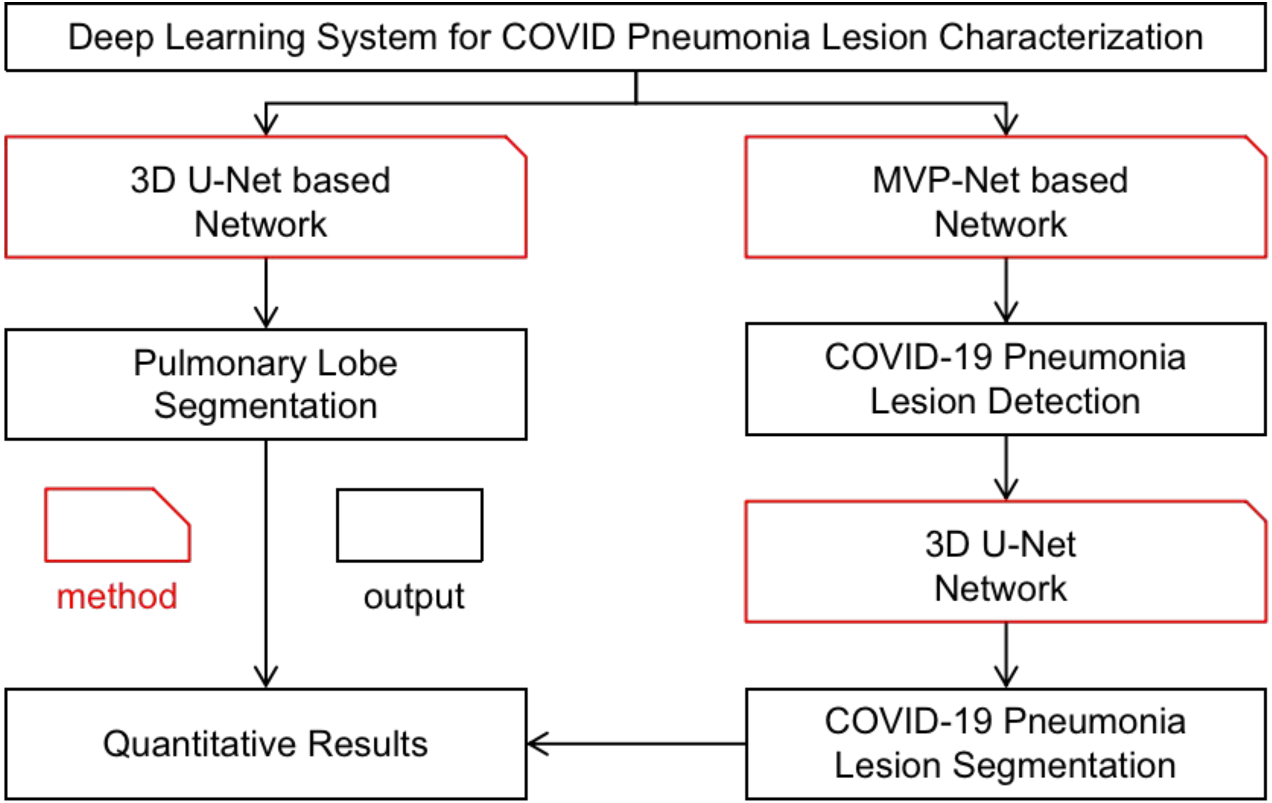

Supplementary Figure 2. Flowchart of the AI system.

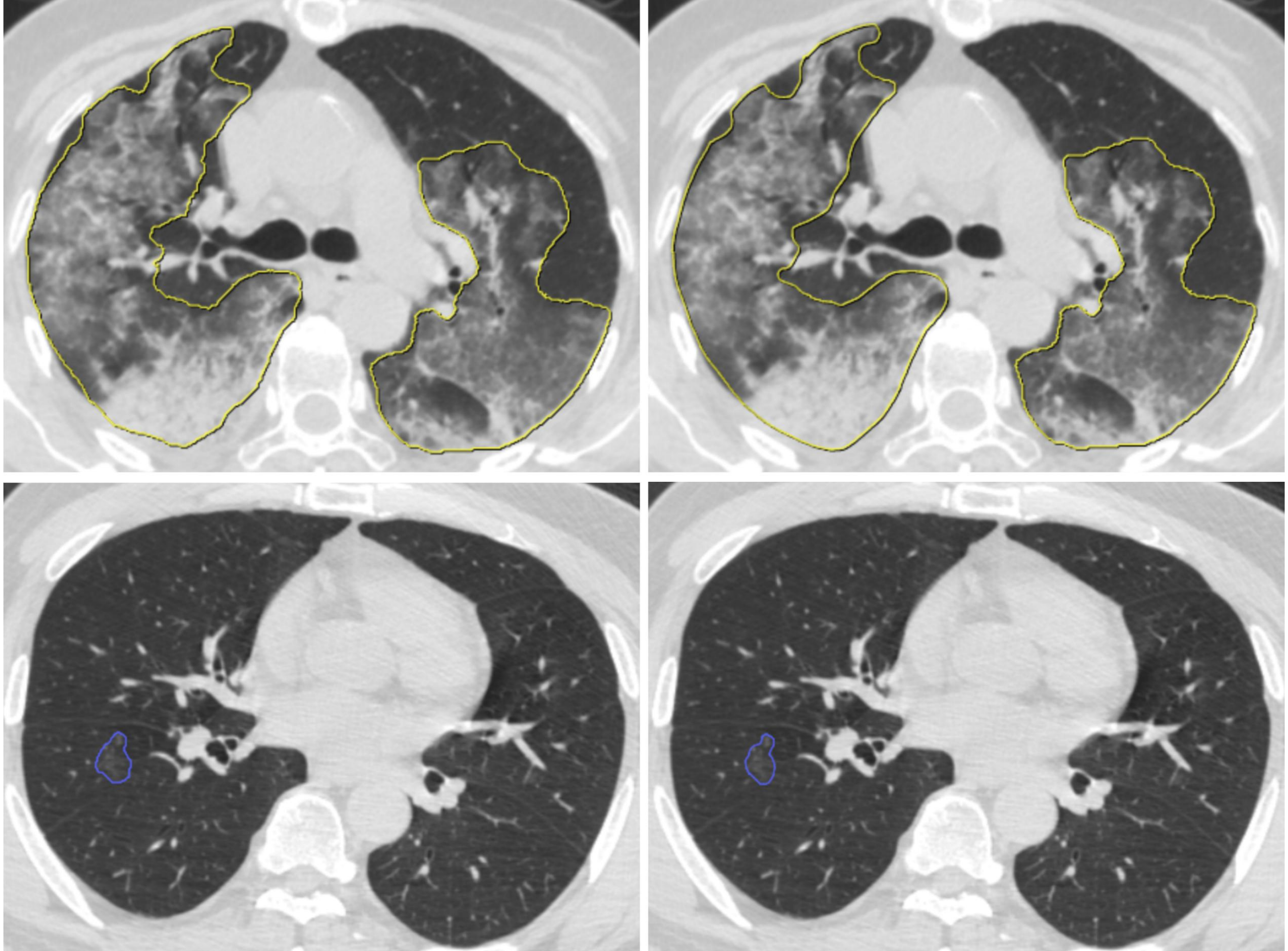

Supplementary Figure 3 Examples of segentations by AI system (left) verse radiologist (right) on the initial CT scan of COVID-19 patients w / o (yellow border and blue border, respectively) severe outcomes

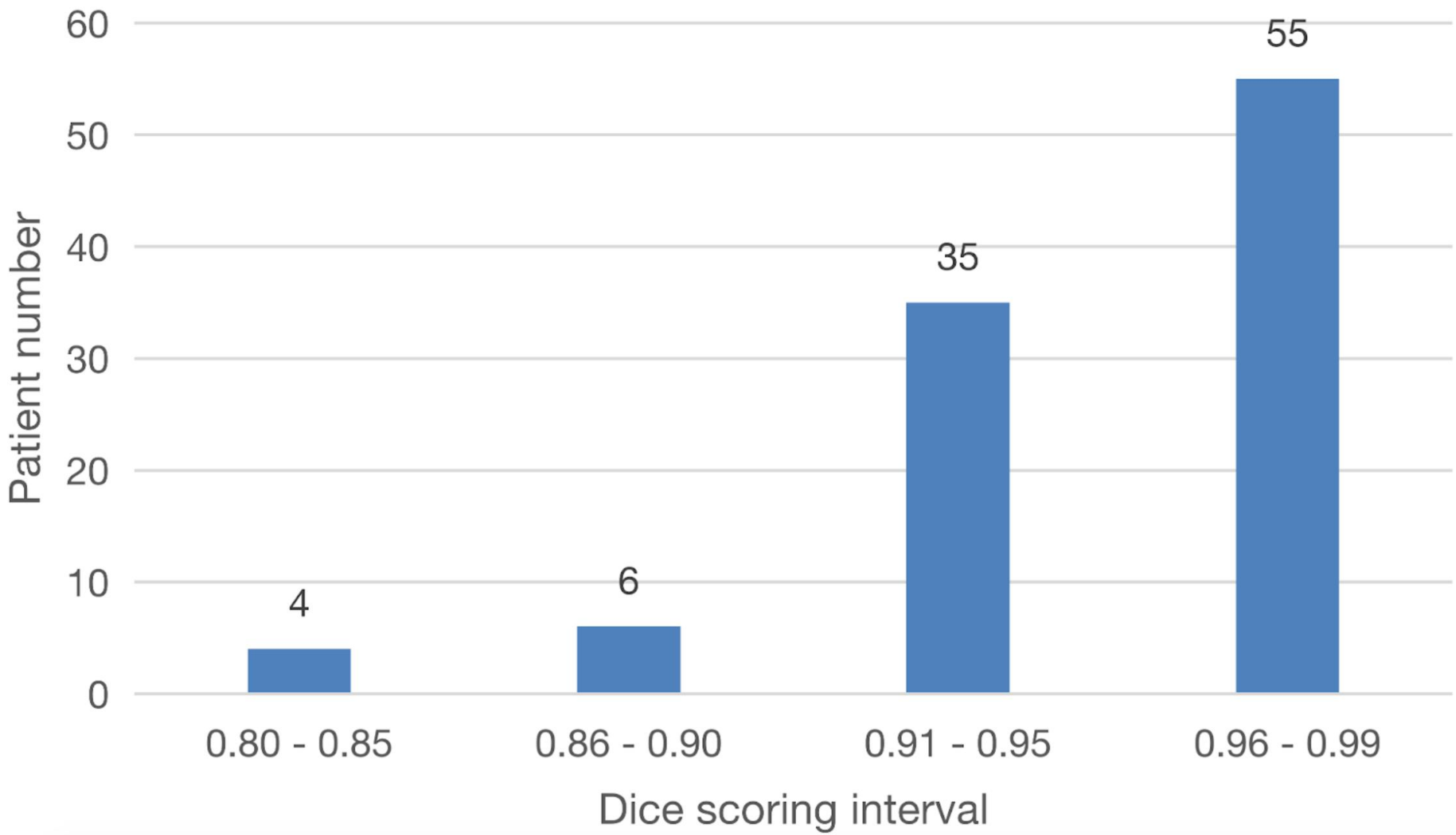

Supplementary Figure 4. The number of patients in different dice scoring intervals

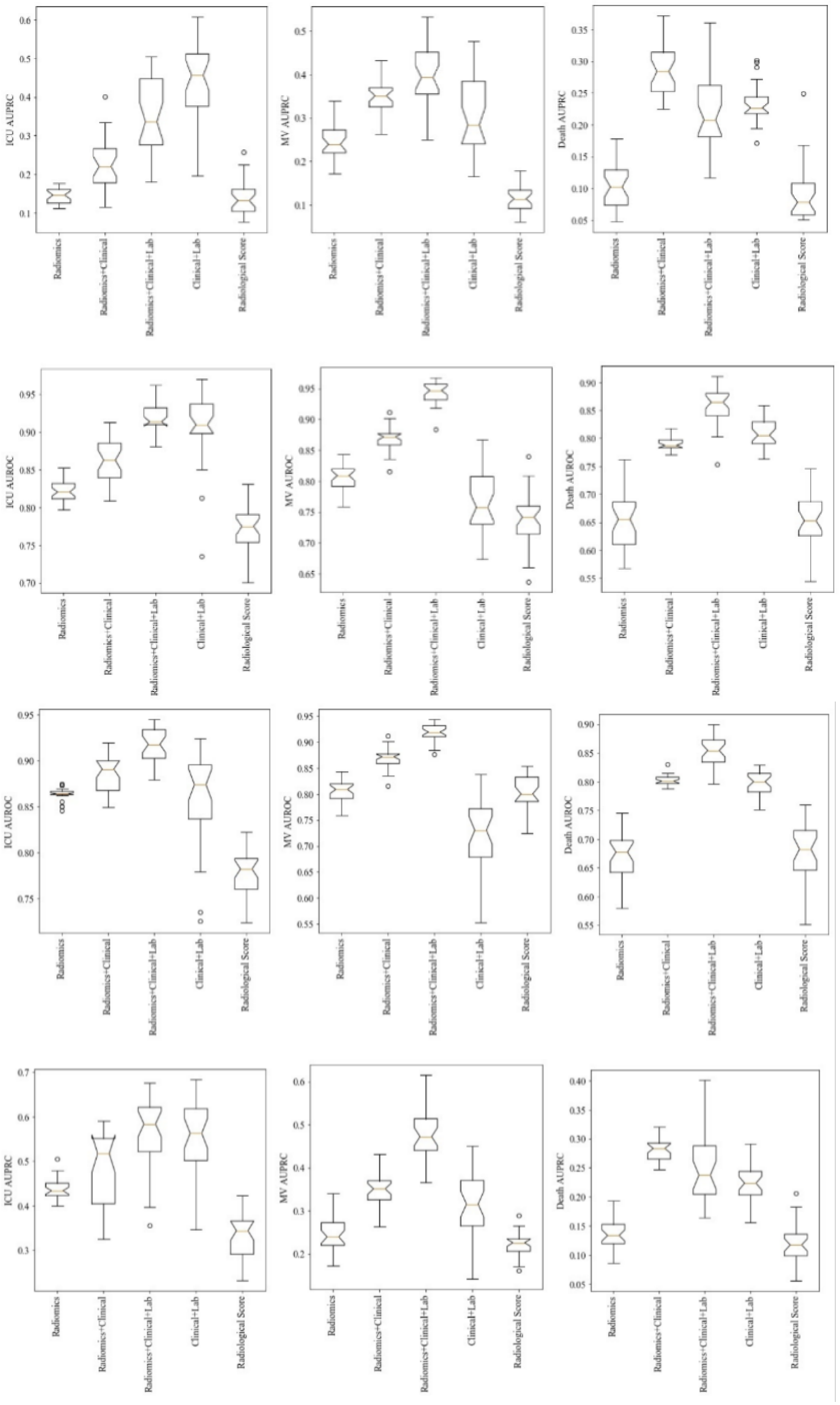

Supplementary Figure 5. The box plots of bootstrapping model performances in terms of AUPRC and AUPRC on three outcome prediction tasks on Cohort 1 (a-f) and Cohort 2 (g-l).

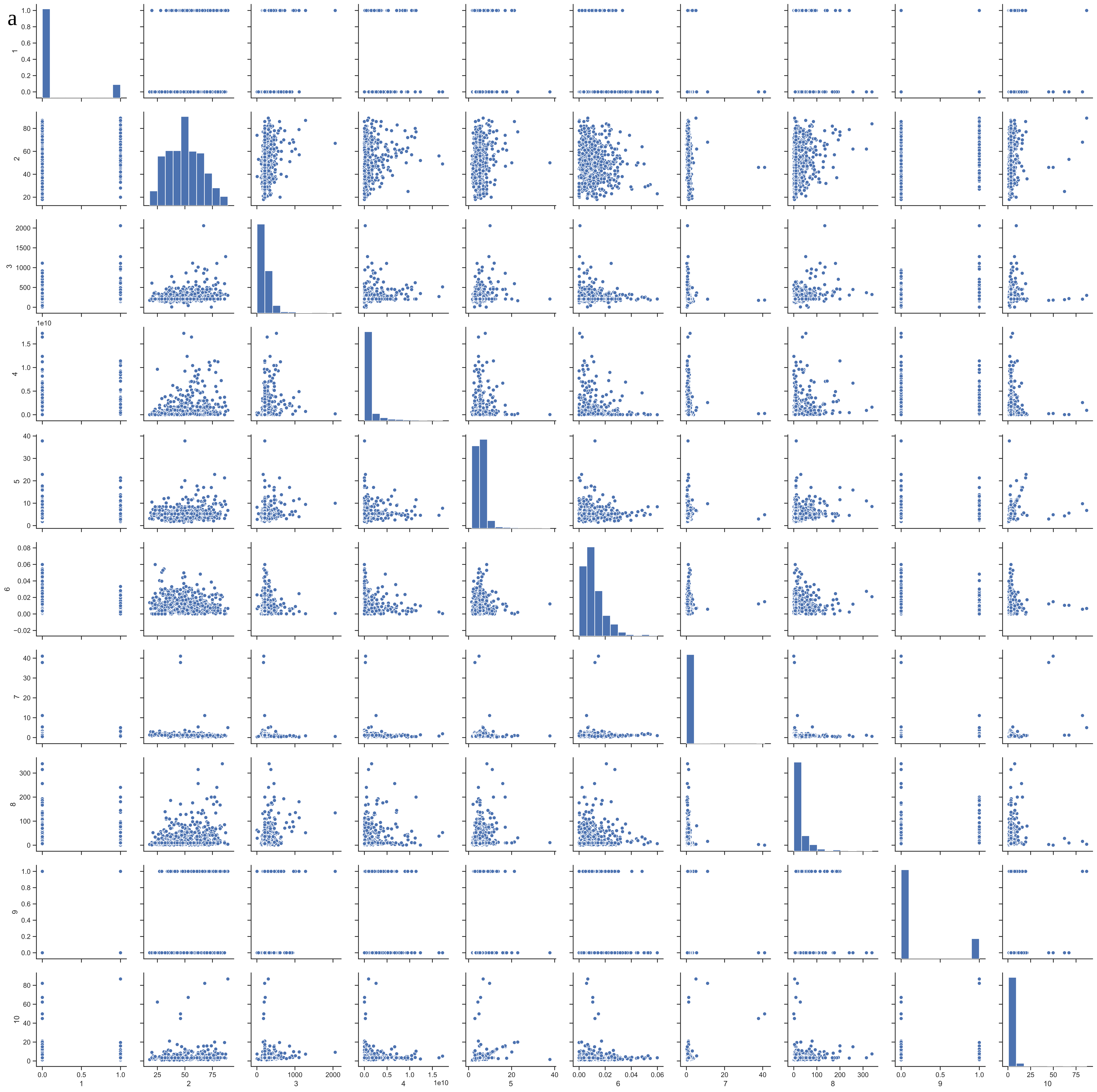
a
1
2
3
4
5
6
7
8
9
10
1e10

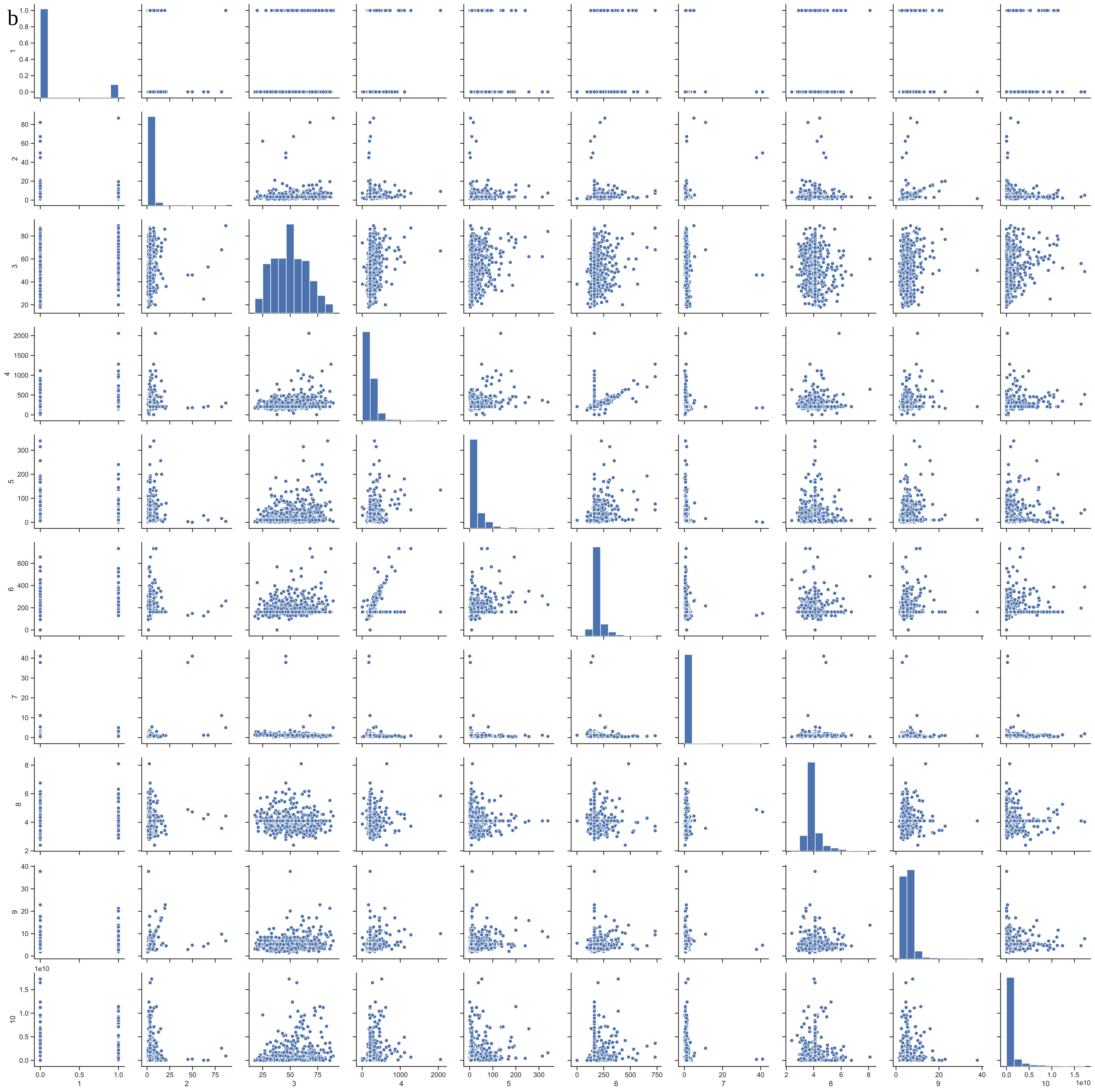
b
1
2
3
4
5
6
7
8
9
10
1e10

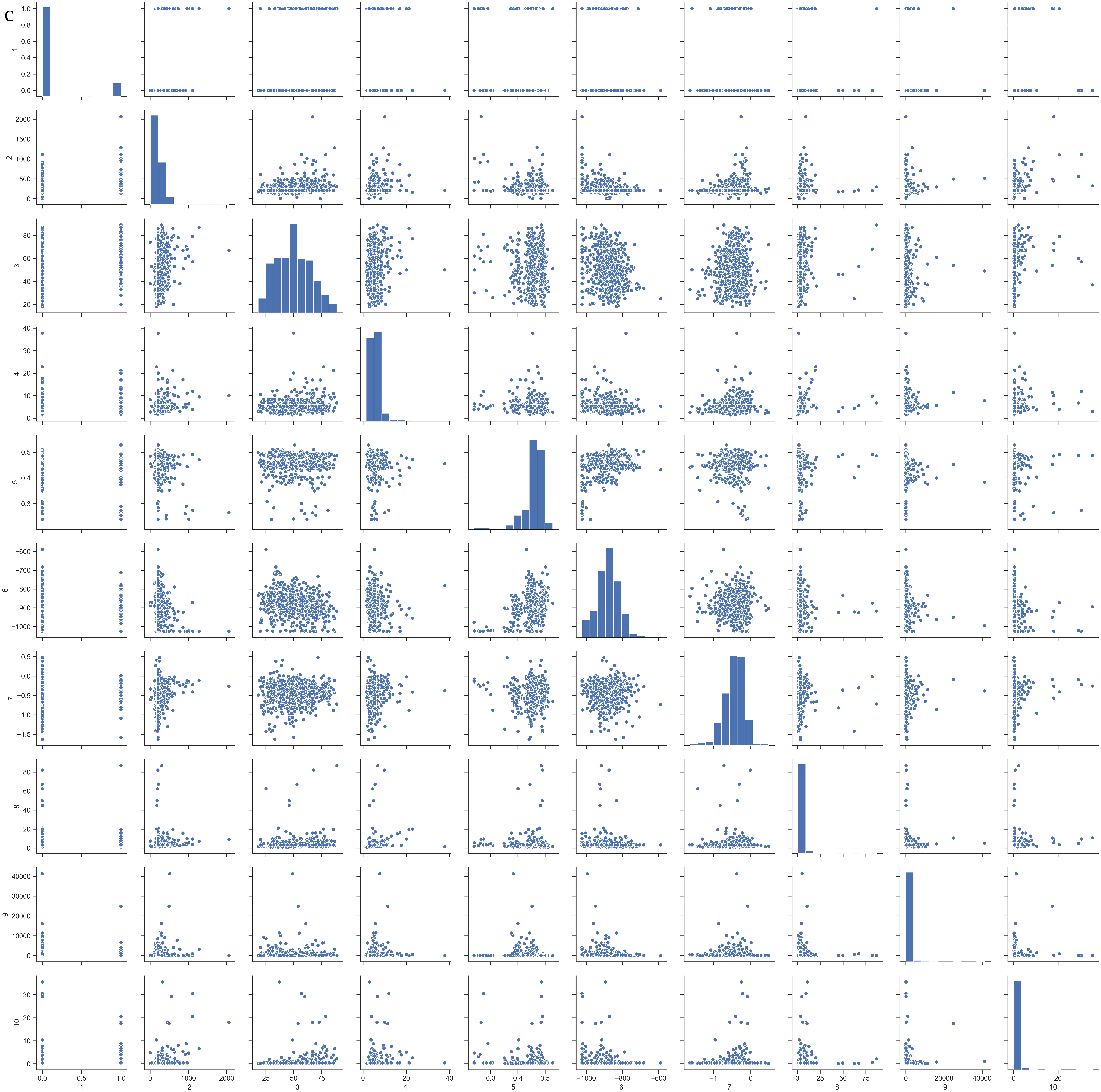
c
1
2
3
4
5
6
7
8
9
10

Supplementary Figure 6 The paired plot of the ten most important features in (a) ICU, (b) MV, (c)death prediction.

Most important features are respectively: (the ranks align with the plots) (a) ICU: 1.Dyspnea, 2.Age, 3.LDH, 4.wavelet-LHH_glszm, 5.WBC, 6.original_glszm, 7.lymphocyte, 8.CRP, 9.hypertension, 10.neutrophil; (b) MV: 1.Dyspnea, 2.Neutrophil, 3.Age, 4.LDH, 5.CRP, 6.HBDH, 7.Lymphocyte, 8.Potassium, 9.WBC, 10.wavelet-LHH_glszm; (c) Death: 1.Dyspnea, 2.LDH, 3.Age, 4.WBC, 5.wavelet-HLH_glcm, 6.original_firstorder, 7.wavelet-LHL_firstorder, 8.Neutrophil, 9. wavelet-HLL_glszm, 10. D-dimer. Plots at the Diagonal positions are histograms of the most important features while plots at non-diagonal positions are scatter plots of a pair of most important features. Each point represents a patient in Cohort 2. No significant correlations are found between radiomics features and clinical features.

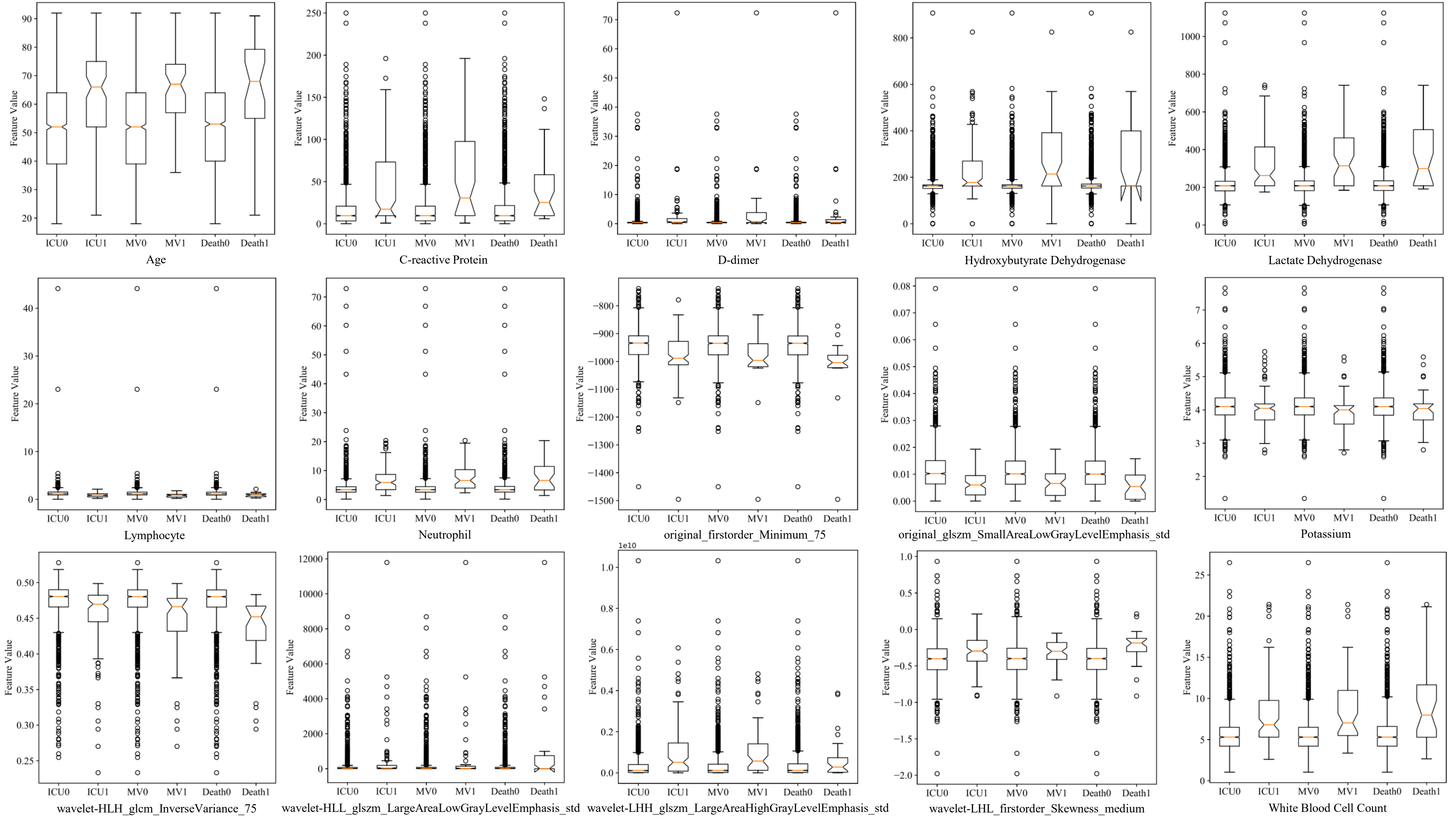
Feature Value
ICU0
ICU1
MV0
MV1
Death0
Death1
Age
C-reactive Protein
D-dimer
Hydroxybutyrate Dehydrogenase
Lactate Dehydrogenase
Lymphocyte
Neutrophil
original_firstorder_Minimum_75
original_glszm_SmallAreaLowGrayLevelEmphasis_std
Potassium
wavelet-HLH_glcm_InverseVariance_75
wavelet-HLL_glszm_LargeAreaLowGrayLevelEmphasis_std
1e10
wavelet-LHH_glszm_LargeAreaHighGrayLevelEmphasis_std
wavelet-LHL_firstorder_Skewness_medium
White Blood Cell Count

Supplementary Figure 7. The feature values of Cohort 1 patients with and without adverse outcomes of the continuous features among all the ten most important features found on three prediction tasks.

The feature values of positive and negative cases of the fifteen continuous variables found among the top ten important features on three tasks (Cohort 1 samples) were shown. The statistical significance was tested and shown (Supplementary Table 7) on the respective ten most important features (both continuous and categorical variables) in ICU prediction, MV prediction and death prediction.

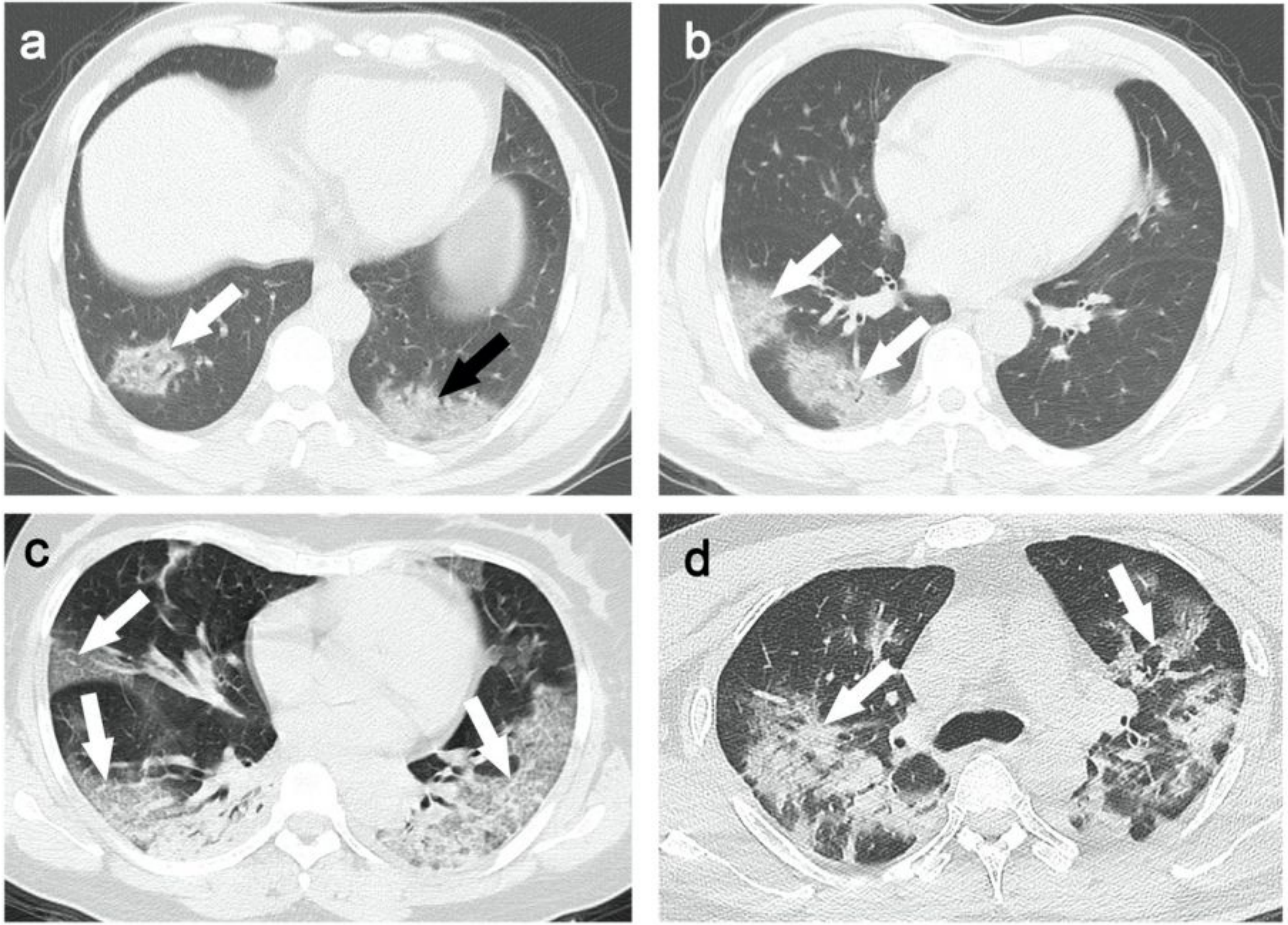
a
b
c
d

Supplementary Figure 8. Examples of semantic features on the initial CT scan of COVID-19 patients with severe outcomes.

(a) Axial CT image obtained without enhancement in a 60-year-old man shows consolidative opacity with a rounded morphology (white arrow) in right lower lobe and shows consolidative opacity with peripheral distribution in left lower lobe (black arrow).

(b) Axial CT image obtained in a 60-year-old man shows consolidative opacities with peripheral distribution in the right lower lobe (arrows).

(c) Axial CT image without intravenous contrast material in a 47-year-old woman shows bilateral ground-glass and consolidative opacities with a striking peripheral distribution (arrows).

(d) Axial CT image obtained in a 39-year-old man shows ground-glass opacities and consolidative opacities with diffuse distribution(arrows).

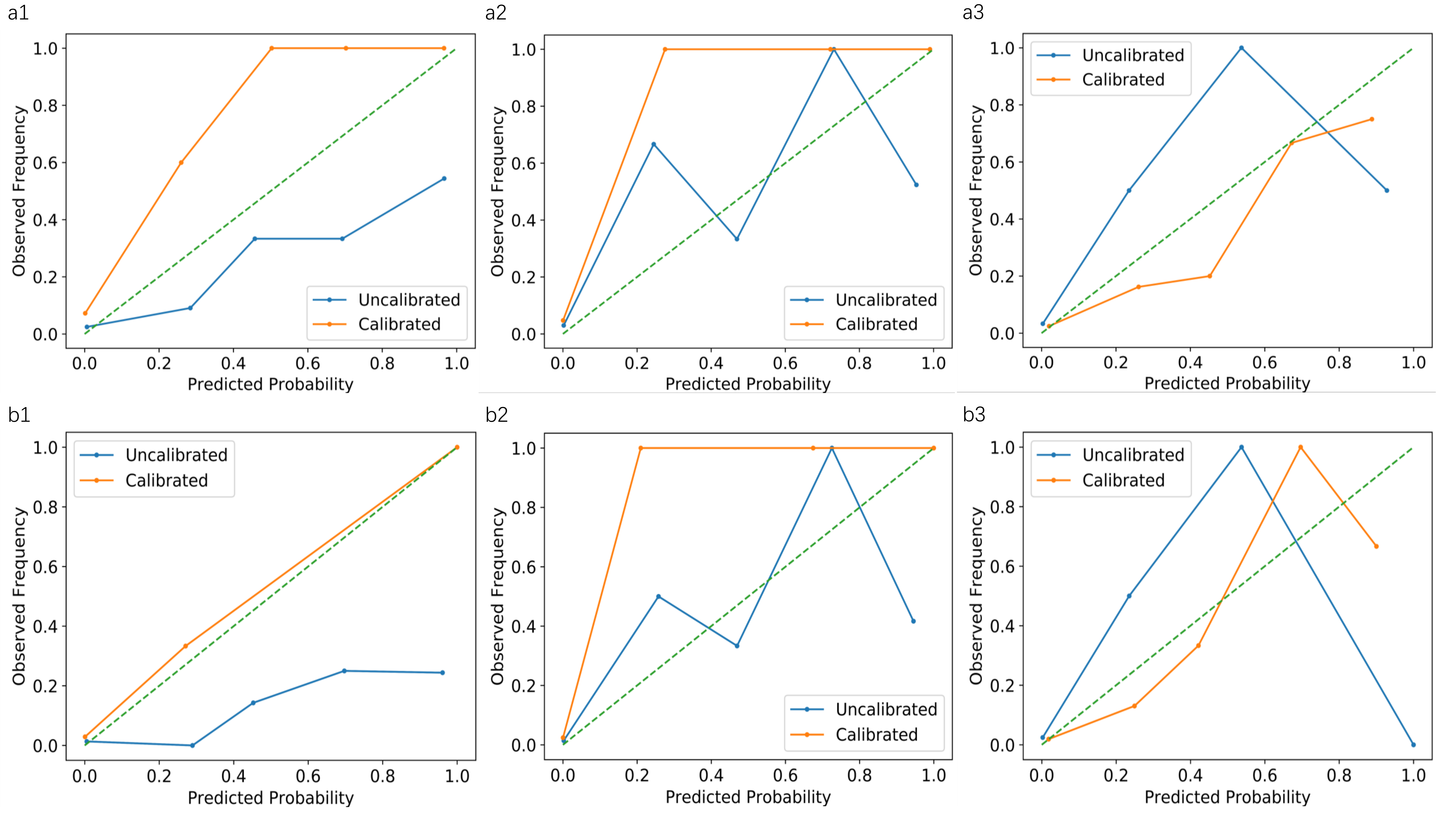

a1
a2
a3
b1
b2
b3
Uncalibrated
Calibrated
Observed Frequency
Predicted Probability
0.0
0.2
0.4
0.6
0.8
1.0

Supplementary Figure 9 The model calibration results of RadioClinLab models on Cohort 2 and Cohort 3.

(a1-a3) Model calibration result of the RadioClinLab ICU prediction model, MV prediction model and death prediction model on Cohort 2.

(b1-b3) Model calibration results of the RadioClinLab ICU prediction model, MV prediction model and death prediction model on Cohort 3.
