# Supplementary material for: AI-based analysis of CT images for rapid triage of COVID-19 patients
Source: NPJ Digit Med. 2021 Apr 22;4:75. doi: 10.1038/s41746-021-00446-z (PMC8062628; doi:10.1038/s41746-021-00446-z)
Supplement: Supplementary file 2 — Reporting Summary [file 41746_2021_446_MOESM2_ESM.pdf]

nature research

Corresponding author(s): Olivier Gevaert, Guangming Lu

Last updated by author(s): Dec 22, 2020

Reporting Summary

Nature Research wishes to improve the reproducibility of the work that we publish. This form provides structure for consistency and transparency in reporting. For further information on Nature Research policies, see our Editorial Policies and the Editorial Policy Checklist.

Statistics

For all statistical analyses, confirm that the following items are present in the figure legend, table legend, main text, or Methods section.

n/a	Confirmed	
☐	☒	The exact sample size (*n*) for each experimental group/condition, given as a discrete number and unit of measurement
☐	☒	A statement on whether measurements were taken from distinct samples or whether the same sample was measured repeatedly
☐	☒	The statistical test(s) used AND whether they are one- or two-sided<br>*Only common tests should be described solely by name; describe more complex techniques in the Methods section.*
☐	☒	A description of all covariates tested
☐	☒	A description of any assumptions or corrections, such as tests of normality and adjustment for multiple comparisons
☐	☒	A full description of the statistical parameters including central tendency (e.g. means) or other basic estimates (e.g. regression coefficient) AND variation (e.g. standard deviation) or associated estimates of uncertainty (e.g. confidence intervals)
☐	☒	For null hypothesis testing, the test statistic (e.g. *F*, *t*, *r*) with confidence intervals, effect sizes, degrees of freedom and *P* value noted<br>*Give P values as exact values whenever suitable.*
☒	☐	For Bayesian analysis, information on the choice of priors and Markov chain Monte Carlo settings
☐	☒	For hierarchical and complex designs, identification of the appropriate level for tests and full reporting of outcomes
☐	☒	Estimates of effect sizes (e.g. Cohen's *d*, Pearson's *r*), indicating how they were calculated

Our web collection on statistics for biologists contains articles on many of the points above.

Software and code

Policy information about availability of computer code

Data collection	The pneumonia was detected and segmented by a deep-learning AI system (Beijing Deepwise & League of PhD Technology Co.Ltd) and Pyradiomics (v3.0) running in the Linux platform was adopted to extract radiomic features. The codes are available here: https://github.com/terryli710/COVID_19_Rapid_Triage_Risk_Predictor.
Data analysis	The codes that support the findings of this study are available here: https://github.com/terryli710/COVID_19_Rapid_Triage_Risk_Predictor. SPSS v15·0 [Chicago, SPSS Inc.] and MedCalc statistical software were used for statistical analysis.

For manuscripts utilizing custom algorithms or software that are central to the research but not yet described in published literature, software must be made available to editors and reviewers. We strongly encourage code deposition in a community repository (e.g. GitHub). See the Nature Research guidelines for submitting code & software for further information.

Data

Policy information about availability of data

All manuscripts must include a data availability statement. This statement should provide the following information, where applicable:

- Accession codes, unique identifiers, or web links for publicly available datasets
- A list of figures that have associated raw data
- A description of any restrictions on data availability

The data that support the findings of this study are available on request from the corresponding author (G.M.L.). The data with participant privacy/consent are not publicly available due to hospital regulation restrictions.

Field-specific reporting

Please select the one below that is the best fit for your research. If you are not sure, read the appropriate sections before making your selection.

☒ Life sciences ☐ Behavioural & social sciences ☐ Ecological, evolutionary & environmental sciences

For a reference copy of the document with all sections, see nature.com/documents/nr-reporting-summary-flat.pdf

Life sciences study design

All studies must disclose on these points even when the disclosure is negative.

Sample size	We collected 3,522 inpatients with laboratory-confirmed SARS-CoV-2 infection from December 27, 2019 to March 31, 2020 from 39 hospitals in China. Data inclusion criteria were as follows: patients received CT examination within 3 days after admission and we had definitive medical records of short-term outcomes such as Intensive Care Unit (ICU), Mechanical Ventilation (MV) therapy, death (defined as the three prediction tasks), or discharge.
Data exclusions	Exclusion criteria due to one of the following:<br>a. Patients age < 18 years old (n = 12)<br>b. Patients transferred to other hospitals or remaining hospitalized without any adverse outcomes (n = 390)<br>c. Patients lack follow-up information (n = 428)<br>d. CT scans with slice thickness > 2.5mm or convolutional kernel not related to lung (n = 322)<br>e. CT scans lack serial information or with motion artifacts or significant resolution reductions (n = 8)
Replication	The AUROC, AUPRC, accuracy value and their 95% CI were listed to assess the model performance. The paired one-sided t-test was used to calculate the statistical significance of the difference between each AUROC and AUPRC value in the bootstrapping experiments. Chi-square test and Fisher's exact test were exploited to compare categorical data while independent t-test and Wilcoxon rank sum test were used to compare the feature values of continuous variables in positive and negative cases in the entire cohort (n = 2362). Proportional test was done to compare the feature values of categorical variables in positive and negative cases among the most important features found by classifiers and test the statistical significance of categorical variables between Cohort 1 and Cohort 2. Kaplan-Meier survival analysis was done on the high-risk and low-risk group based on predictions and log-rank test was used to evaluate statistical significance.
Randomization	A total of 2362 patients from 39 hospitals were used in this study. We randomly selected 17 hospitals as a primary cohort (Cohort 1, n = 1662) for model development, and the data were split into training and testing sets (ratio 7:3) using stratified random sampling based on death cases in this cohort. Data form other hospitals was used as a validation cohort (Cohort 2, n = 700). The models are available here: https://github.com/terryli710/COVID_19_Rapid_Triage_Risk_Predictor", which includes the code for "Environment building'', "Feature extraction pipeline" and "Prediction models Pipeline" with a DOI: 10.5281/zenodo.4323806.
Blinding	The investigators were blinded to group allocation during data collection and analysis. We collected laboratory-confirmed SARS-CoV-2 inpatients with clear medical records and complete CT data from December 27, 2019 to March 31, 2020. Also, the training set and test sets were randomly divided when the model is developed and validated.

Reporting for specific materials, systems and methods

We require information from authors about some types of materials, experimental systems and methods used in many studies. Here, indicate whether each material, system or method listed is relevant to your study. If you are not sure if a list item applies to your research, read the appropriate section before selecting a response.

Materials & experimental systems

n/a	Involved in the study	
☒	☐	Antibodies
☒	☐	Eukaryotic cell lines
☒	☐	Palaeontology and archaeology
☒	☐	Animals and other organisms
☐	☒	Human research participants
☒	☐	Clinical data
☒	☐	Dual use research of concern

Methods

n/a	Involved in the study	
☒	☐	ChIP-seq
☒	☐	Flow cytometry
☒	☐	MRI-based neuroimaging

Human research participants

Policy information about studies involving human research participants

Population characteristics	(a) demographics: age and gender; (b) comorbidities: coronary heart disease, diabetes, hypertension, chronic obstructive lung disease (COPD), chronic liver disease, chronic kidney disease, and carcinoma; and (c) clinical symptoms: fever, cough, myalgia, fatigue, headache, nausea or vomiting, diarrhea, abdominal pain, and dyspnea.

Recruitment	We collected 3,522 inpatients with laboratory-confirmed SARS-CoV-2 infection from December 27, 2019 to March 31, 2020 from 39 hospitals in China. Data inclusion criteria were as follows: patients received CT examination within 3 days after admission and we had definitive medical records of short-term outcomes such as Intensive Care Unit (ICU), Mechanical Ventilation (MV) therapy, death (defined as the three prediction tasks), or discharge. Our data was only from Chinese hospitals which could potentially limit the generalizability of models in other areas.
Ethics oversight	The protocol of this multi-center study was approved by the institutional review board of Jinling Hospital, Nanjing University School of Medicine (2020NZKY-005-02).

Note that full information on the approval of the study protocol must also be provided in the manuscript.
